# Expression of different L1 isoforms of *Mastomys natalensis* papillomavirus as mechanism to circumvent adaptive immunity

**DOI:** 10.7554/eLife.57626

**Published:** 2020-08-04

**Authors:** Yingying Fu, Rui Cao, Miriam Schäfer, Sonja Stephan, Ilona Braspenning-Wesch, Laura Schmitt, Ralf Bischoff, Martin Müller, Kai Schäfer, Sabrina E Vinzón, Frank Rösl, Daniel Hasche

**Affiliations:** 1 Division of Viral Transformation Mechanisms, Research Program 'Infection, Inflammation and Cancer', German Cancer Research CenterHeidelbergGermany; 2 Division of Functional Genome Analysis, Research Program 'Functional and Structural Genomics', German Cancer Research CenterHeidelbergGermany; 3 Research Group Tumorvirus-specific Vaccination Strategies, Research Program 'Infection, Inflammation and Cancer', German Cancer Research CenterHeidelbergGermany; University of CambridgeUnited Kingdom; Indian Institute of Science Education and Research (IISER)India

**Keywords:** Mastomys coucha, papillomavirus, immune escape, seroconversion, neutralization, virus-like particle, Other

## Abstract

Although many high-risk mucosal and cutaneous human papillomaviruses (HPVs) theoretically have the potential to synthesize L1 isoforms differing in length, previous seroepidemiological studies only focused on the short L1 variants, co-assembling with L2 to infectious virions. Using the multimammate mouse *Mastomys coucha* as preclinical model, this is the first study demonstrating seroconversion against different L1 isoforms during the natural course of papillomavirus infection. Intriguingly, positivity with the cutaneous MnPV was accompanied by a strong seroresponse against a longer L1 isoform, but to our surprise, the raised antibodies were non-neutralizing. Only after a delay of around 4 months, protecting antibodies against the short L1 appeared, enabling the virus to successfully establish an infection. This argues for a novel humoral immune escape mechanism that may also have important implications on the interpretation of epidemiological data in terms of seropositivity and protection of PV infections in general.

## eLife digest

Cancer is not one disease but rather a collection of disorders. As such there are many reasons why someone may develop cancer during their lifetime, including the individual’s family history, lifestyle and habits. Infections with certain viruses can also lead to cancer and human papillomaviruses are viruses that establish long-term infections that may result in cancers including cervical and anal cancer, and the most common form of cancer worldwide, non-melanoma skin cancer.

The human papillomavirus, or HPV for short, is made up of DNA surrounded by a protective shell, which contains many repeats of a protein called L1. These L1 proteins stick to the surfaces of human cells, allowing the virus to get access inside, where it can replicate before spreading to new cells. The immune system responds strongly to HPV infections by releasing antibodies that latch onto L1 proteins. It was therefore not clear how HPV could establish the long-term infections and cause cancer when it was seeming being recognized by the immune system.

Now, Fu et al. have used the Southern multimammate mouse, *Mastomys coucha*, as a model system for an HPV infection to uncover how papillomaviruses can avoid the immune response. This African rodent is naturally infected with a skin papillomavirus called MnPV which, like its counterpart in humans, can trigger the formation of skin warts and malignant skin tumors.

Fu et al. took blood samples from animals that had been infected with the virus over a period of 76 weeks to monitor their immune response overtime. This revealed that, in the early stages of infection, the virus made longer-than-normal versions of the L1 protein. Further analysis showed that these proteins could not form the virus’s protective shell but could trigger the animals to produce antibodies against them. Fu et al. went on to show that the antibodies that recognized the longer variants of L1 protein where “non-neutralizing”, meaning that could not block the spread of the virus, which is a prerequisite for immunity. It was only after a delay of four months that the animals started making neutralizing antibodies that were directed against the shorter L1 proteins that actually makes up the virus’s protective coat.

These findings suggest that virus initially uses the longer version of the L1 protein as a decoy to circumvent the attention of the immune system and provide itself with enough time to establish an infection. The findings also have implications for other studies that have sought to assess the success of an immune response during a papillomavirus infection. Specifically, the delayed production of the neutralizing antibodies means that their presence does not necessarily indicate that a patient is not already infected by a papillomavirus that in the future may cause cancer.

## Introduction

Human Papillomaviruses (HPVs) are widely distributed in nature and more than 220 types were sequenced up to date (PaVE: Papillomavirus Episteme). They cannot only be divided in mucosal and cutaneous types (Bzhalava et al., 2013), but also whether the infection is acquired, for example via sexual intercourse (as for high-risk genital HPVs) (Gravitt and Winer, 2017) or whether a commensal cohabitation (as for cutaneous HPVs) occurred shortly after birth (Antonsson et al., 2003; Weissenborn et al., 2009). Depending on environmental factors (e.g. chronic UV exposure) (Rollison et al., 2019; Uberoi et al., 2016), the individual immune status (e.g. systemic immunosuppression) (Reusser et al., 2015; Vinzón and Rösl, 2015) or genetic predispositions (e.g. EVER1/2 mutations in *Epidermodysplasia verruciformis* patients) (de Jong et al., 2018), commensal cutaneous papillomaviruses can induce hyperproliferative lesions (e.g. actinic keratosis) which may progress to squamous cell carcinomas (SCCs) (Hasche et al., 2018).

The African multimammate rodent *Mastomys coucha* represents a unique model system to investigate the consequences of a natural PV infection in the context of skin carcinogenesis (Hasche and Rösl, 2019). The animals become infected with *Mastomys natalensis* papillomavirus (MnPV) soon after birth (Schäfer et al., 2011) and seroconversion against viral proteins can be detected shortly afterwards (Schäfer et al., 2010). MnPV is a typical cutaneous PV that resembles human β-types by lacking an E5 open-reading frame (ORF) (Tan et al., 1994). Characterization of the viral transcriptome in productive lesions revealed a complex splicing pattern with different promoters and transcriptional start sites (Salvermoser et al., 2016), also described for some HPV types (Sankovski et al., 2014; Wang et al., 2011) or for the mouse papillomavirus type 1 (MmuPV1) (Xue et al., 2017). Most of these transcripts are polycistronic, allowing (at least hypothetically) the translation of several different ORFs (Salvermoser et al., 2016).

Using *Mastomys coucha* as a preclinical model, we could show that immunization with MnPV virus-like-particles (VLPs) induces a long-lasting neutralizing antibody response that completely prevents the appearance of skin lesions both under immunocompetent and immunosuppressed conditions (Vinzón et al., 2014). Furthermore, *Mastomys coucha* also represents a paradigm for SCC development in the context of MnPV infection and UV exposure, thereby reflecting many aspects found in humans where a ‘hit-and-run’ mechanism during carcinogenesis is supposed (Hasche et al., 2017; Hasche et al., 2018).

Virions of PVs consist of 72 pentamers of the major (L1) protein together with up to 72 molecules of the minor (L2) capsid protein (Buck et al., 2013; Hagensee et al., 1993; Wang and Roden, 2013). The L1 protein has the capability to spontaneously form regular structures (capsomers), triggered by a thermodynamically favored self-assembly process (McManus et al., 2016). Due to their repetitive structures, PV particles are very immunogenic and induce the generation of neutralizing antibodies that block viral entry into the host cell via binding to conformational epitopes on the capsid (Kwak et al., 2011; Wang and Roden, 2013).

Considering the cross-talk between viral infections and the immune system, PVs have developed multiple strategies to escape from immune surveillance (Bordignon et al., 2017). While there is plenty of information about how innate immunity as the first line of defense is circumvented (Christensen, 2016; Smola et al., 2017), less is known about the humoral immune response in terms of generation of protecting antibodies during the natural course of a PV infection.

In the present study, we show that MnPV, as a rodent equivalent for cutaneous PVs in humans, induces a strong seroconversion in its natural host early after infection. However, the raised antibodies are non-neutralizing and directed against a longer isoform of the L1 protein which is unable to assemble into viral particles. Only after a delay of around 4 months after infection, protecting antibodies appear. This argues for a novel PV immune escape mechanism, probably providing a selective advantage to establish an efficient infection. We characterized this mechanism in greater detail since it may also have important implications in understanding the humoral immune response during a normal infection cycle in general.

## Results

### Alternative translation initiation codons of the PV L1 ORF

**Figure 1.**
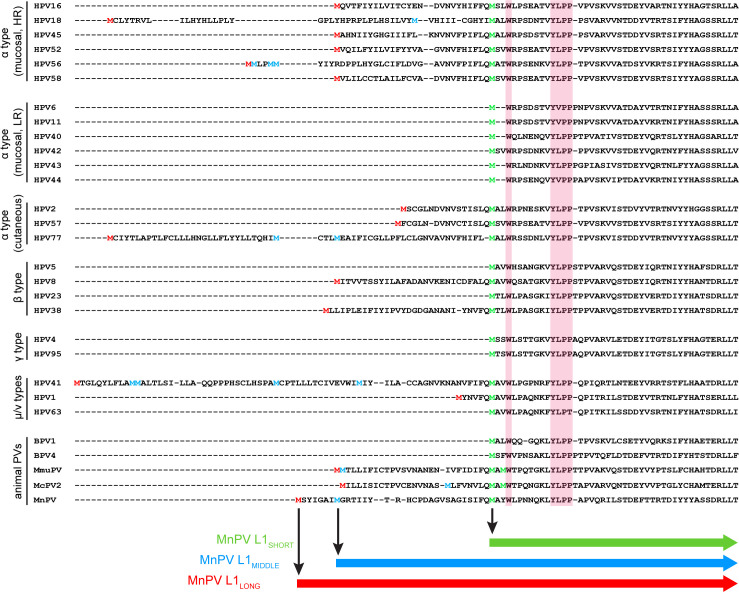
Alignment of L1 sequences from different PV types. N-terminal sequences of L1 proteins from 29 PV types were aligned using Clustal Omega. The highly conserved motif (Wx_7_YLPP) is marked in pink boxes. The first methionine of L1 is marked in red. The last methionine upstream of the Wx_7_YLPP motif is shown in green and methionines between the first and the last one are depicted in blue. In the case of MmuPV and McPV2, both methionines upstream of the conserved motif fit to the consensus sequence of L1_SHORT_ and are therefore depicted in green.

Based on two previous studies comparing the presence of initiation codons within the papillomavirus L1 ORF (Joh et al., 2014; Webb et al., 2005), their position was aligned according to the PV genera derivation (Bzhalava et al., 2015; Van Doorslaer et al., 2013; Figure 1). Notably, alternative ATGs can be found in various mucosal ‘high-risk’ HPV types such as 16, 18, 45, 52, 56, 58, but not in ‘low-risk’ types such as HPV6, 11, 40, 42, 43, 44, respectively (Webb et al., 2005). Additional in-frame initiation codons can also be detected in cutaneous HPV types of several genera such as HPV1, 2, 8, 38, 41, 57 and 77, respectively, of which HPV8 and HPV38 are considered to be ‘high-risk’ cutaneous HPVs (Rollison et al., 2019; Tommasino, 2017). Accordingly, due to the presence of potential alternative translation initiation sites, different L1 isoforms could be translated. As shown in Figure 1, almost all outlined PV L1 proteins harbor a consensus Wx_7_YLPP motif within the N-terminal region (Joh et al., 2014), independently from PV genus or cancer risk assessment, while the remaining N-terminal sequences are not very conserved. For the majority of PV types, the nearest methionine codon to this motif is located one to three amino acids upstream of the tryptophan (W) in the consensus motif. However, there are also exceptions from this rule since HPV31, 35 and 51, for instance, harbor one additional ATG followed by an interspersed in-frame TAA stop codon, thereby probably preventing the synthesis of additional L1 isoforms (Webb et al., 2005). Intriguingly, the MnPV L1 ORF also contains three alternative ATGs, which are located at nucleotide positions (nt) 5704, 5725 and 5797, potentially leading to the expression of L1_LONG_, L1_MIDDLE_ and L1_SHORT_ proteins, respectively.

### Anti-L1_LONG_ and anti-L1_SHORT_ seroresponses emerge at different time points after infection

**Figure 2.**
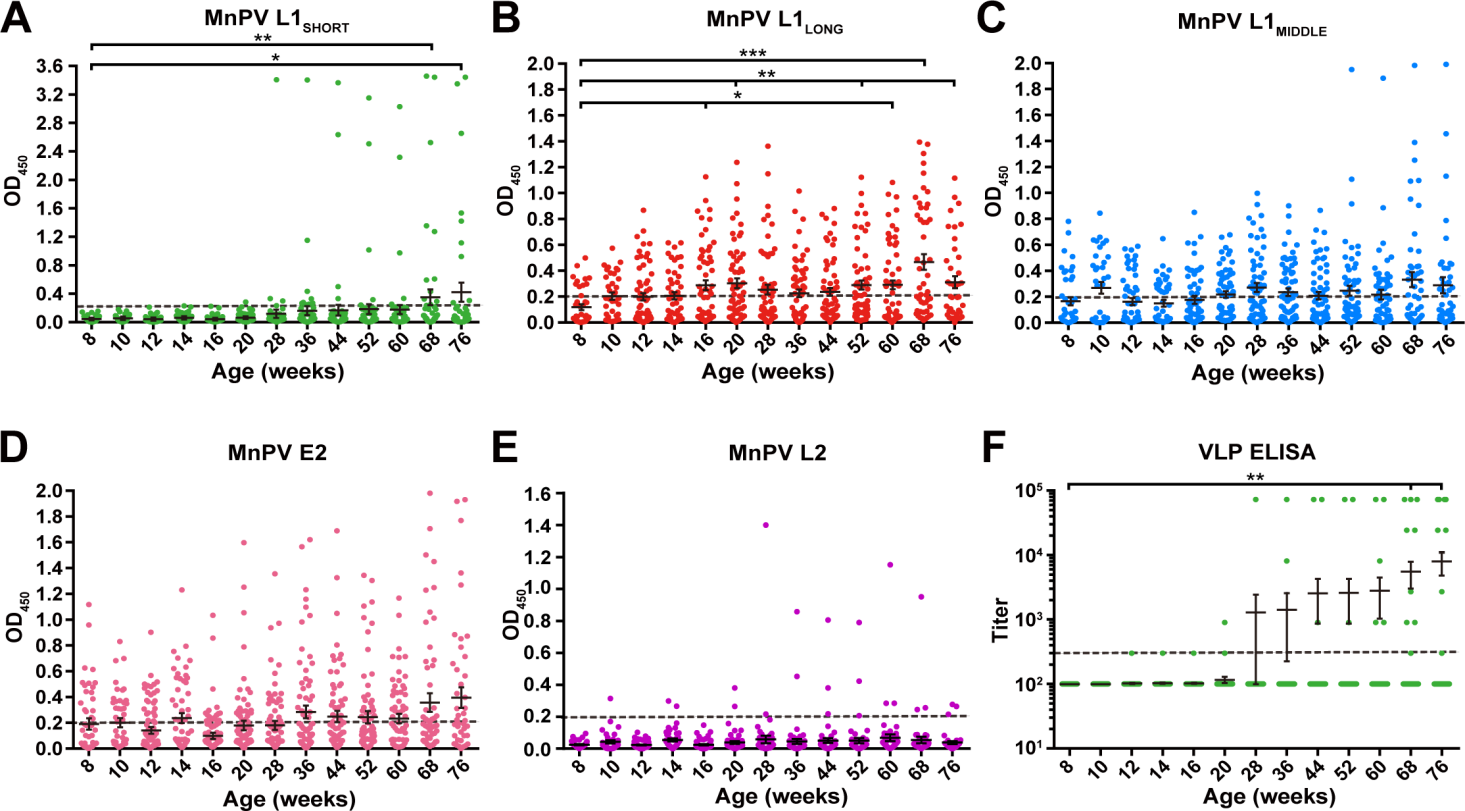
Seroreactivity against viral proteins in naturally MnPV-infected animals. Seroresponses of 682 sera from 60 animals measured by GST-ELISA against (**A**) L1_SHORT_, (**B**) L1_LONG_, (**C**) L1_MIDDLE_, (**D**) E2 and (**E**) L2 GST-fusion proteins and F) VLP-ELISA. Dashed lines represent the methods’ cut-off (OD_450_ = 0.2 for GST-ELISA or titer of 300 for VLP-ELISA) (Mean ± SEM; 1-Way-ANOVA test, *p<0.05, **p<0.01, ***p<0.001).

To examine serological responses against the three putative MnPV L1 isoforms, 60 naturally infected animals were monitored during different stages of viral infection (682 sera in total) encompassing an age between 8 and 76 weeks. Since most serological detection methods developed to date are based on the L1_SHORT_ isoform, firstly we examined seroconversion against L1_SHORT_ by glutathione S-transferase (GST)-capture ELISA (Kricker et al., 2020; Sehr et al., 2002; Waterboer et al., 2009). Notably, only few animals (8/60) exhibited measurable seroresponses against L1_SHORT,_ initially at an age of 28 weeks. The mean seroreactivity per time point exceeded the cut-off earliest at an age of 68 weeks (Figure 2A). Conversely, in 27.5% of the animals, broad seroresponses against MnPV L1_LONG_ were already detectable as early as 8 weeks of age which increased to 52.5% of the animals at 76 weeks (Figure 2B). In the main comparison at the latest time point where most animals were still alive (68 weeks), a significant difference was observed (p<0.001, two-tailed McNemar’s test, see Figure 2—figure supplement 1). Seroreactivity against L1_MIDDLE_ shows a similar time course as L1_LONG_ which is consistent with the correlation between both (Figure 2C and Figure 2—figure supplement 2. Seroresponses against the E2 protein, which is involved in viral DNA replication (McBride, 2013) and considered as an early marker of infection (Schäfer et al., 2011), developed shortly after birth and increased during the study (Figure 2D). Conversely, seroconversion against the minor capsid protein L2 appeared only in a few of the animals and as late as seroconversion against L1_SHORT_ (Figure 2E).

**Figure 2—figure supplement 1.**
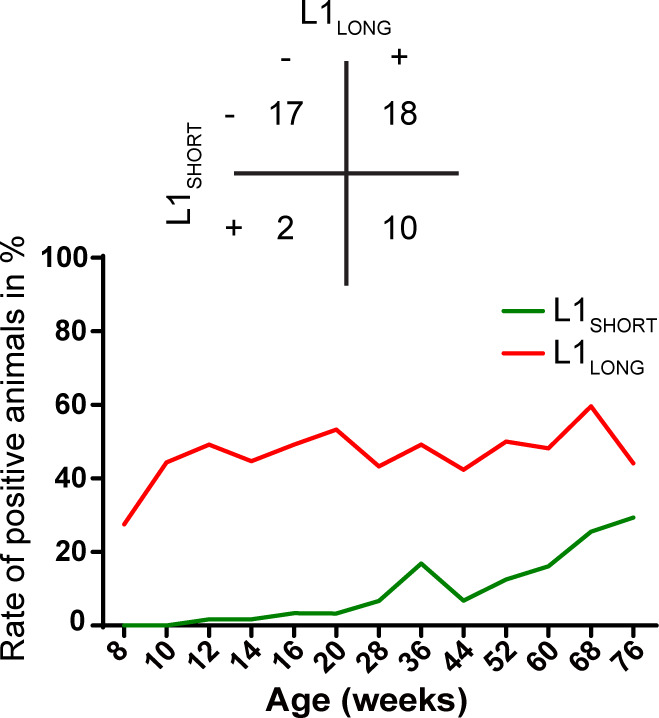
Rate of L1_LONG_ and L1_SHORT_ positive animals. Rate of L1_LONG_ and L1_SHORT_ positive animals at different time points of the follow-up study. The 2 × 2 contingency table used for the two-tailed McNemar’s test shows the 68 week time point, which was used for the main comparison, since here, most animals were still alive.

**Figure 2—figure supplement 2.**
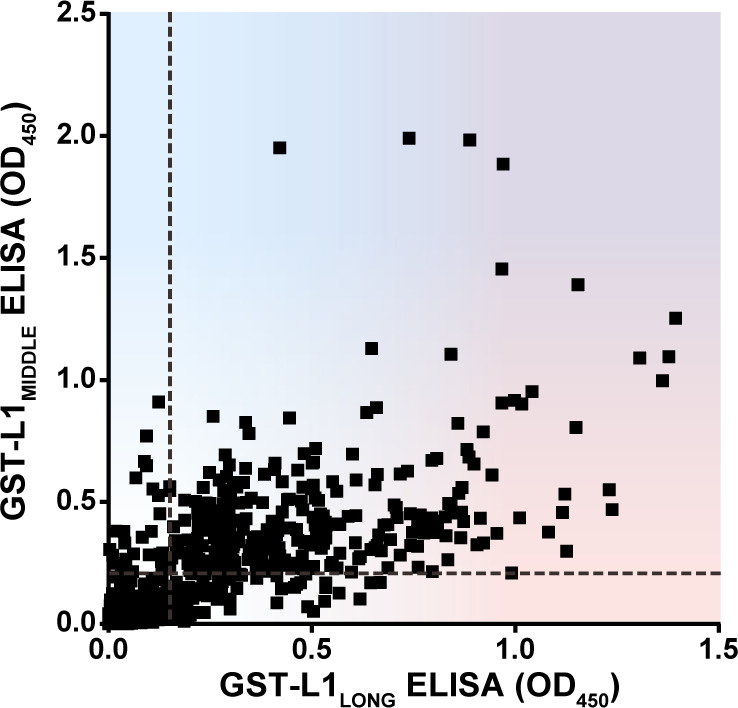
Additional correlation of seroreactivities measured by GST-ELISA. Correlation of seroreactivities against GST-L1_LONG_ with seroreactivities against GST-L1_MIDDLE_ (correlation coefficient, R^2^ = 0.4723).

**Figure 3.**
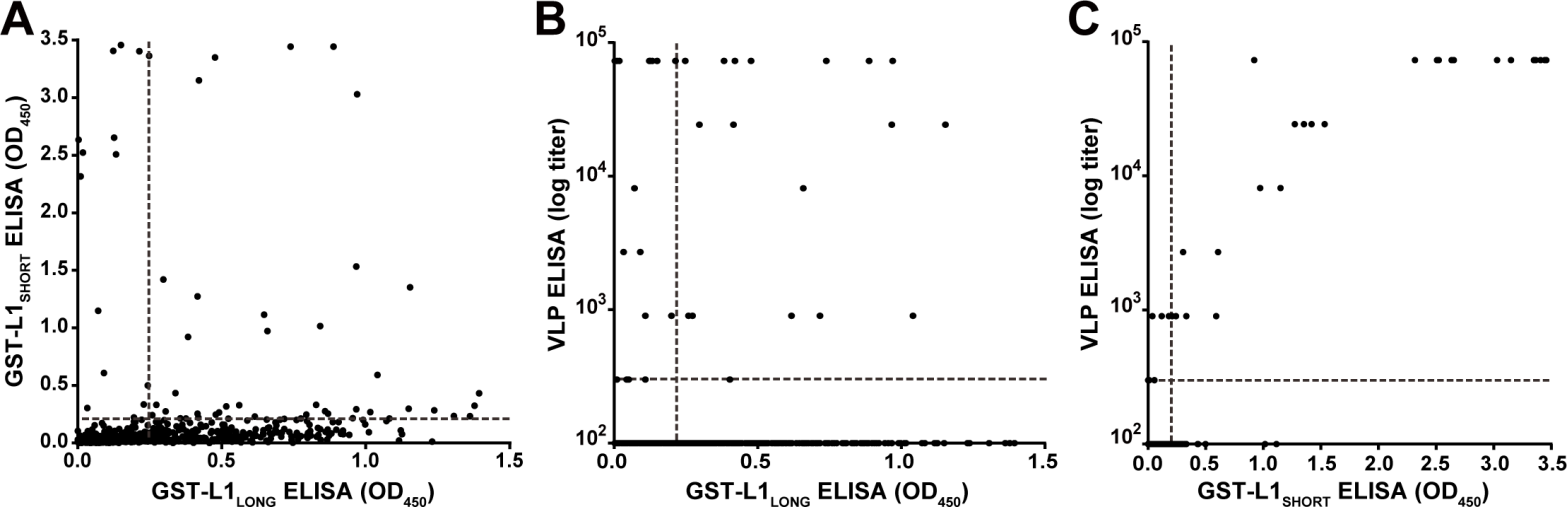
Correlation of GST- and VLP-ELISAs. (**A**) Correlation of seroreactivities against GST-L1_LONG_ with seroreactivities against GST-L1_SHORT_ (correlation coefficient, R^2^ = 0.0261). (**B**) Correlation of GST-L1_LONG_ ELISA with VLP-ELISA (R^2^ = 0.0062). (**C**) Correlation of GST-L1_SHORT_ ELISA with VLP-ELISA (R^2^ = 0.8394). All graphs include all 682 sera taken during the study. Dashed lines indicate the methods’ cut-offs.

In order to exclude possible experimental bias merely working with a GST-fusion protein-based L1_SHORT_ ELISA, additional ELISAs were performed using MnPV VLPs (derived from L1_SHORT_) produced via baculovirus expression system (Christensen et al., 1994; Rose et al., 1993). In accordance with the GST-L1_SHORT_ ELISA, serum responses against VLPs were absent in early infection stages and did not exceed the cut-off before an age of 20 weeks (Figure 2F). This suggests that anti-L1_LONG_ antibodies during early infection fail to recognize epitopes of both GST-L1_SHORT_ antigen and on the surface of intact VLPs. Accordingly, there is no correlation of anti-GST-L1_LONG_ with anti-GST-L1_SHORT_ or anti-VLP reactivity (Figure 3A and B). Conversely, a significant correlation between GST-L1_SHORT_ and VLP-ELISA (Figure 3C) strengthens the notion that the absence of a correlation between GST-L1_LONG_ and L1_SHORT_ ELISAs was indeed due to altered serological properties of L1 isoforms rather than due to different ELISA methodologies.

### Anti-L1_LONG_ antibodies lack neutralizing capacity

**Figure 4.**
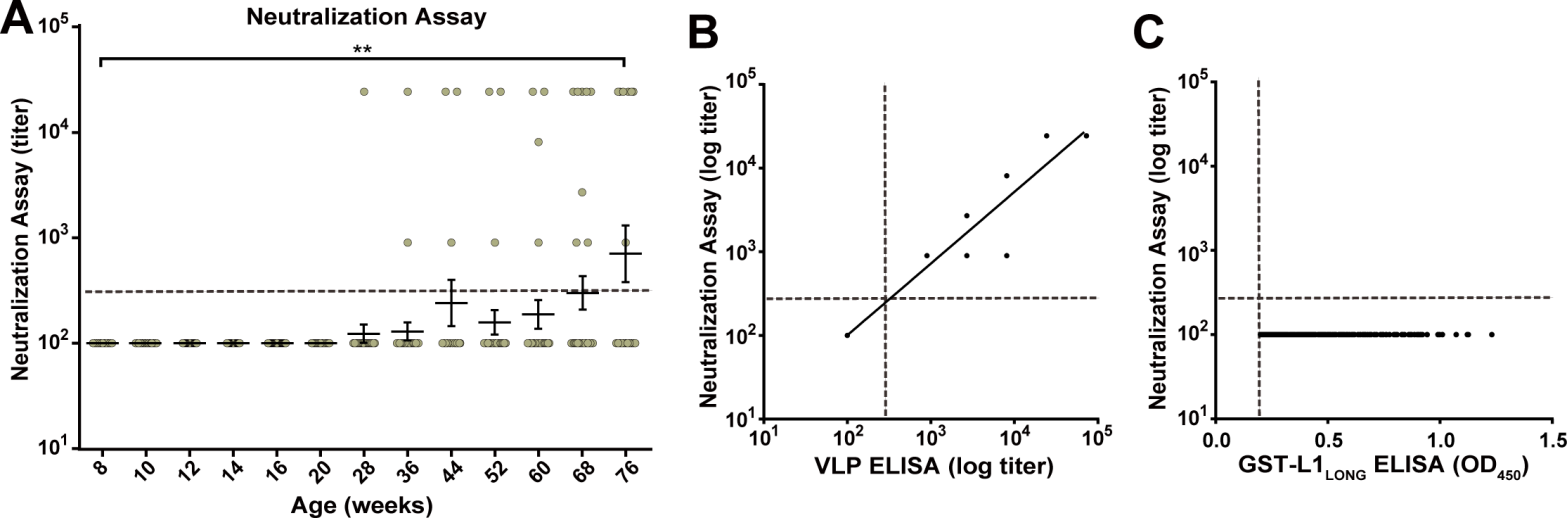
Neutralizing capacity of anti-L1_LONG_ and anti-L1_SHORT_ antibodies. (**A**) Neutralization assay for all L1_LONG_-/L1_SHORT_-positive sera (n = 294) from 60 naturally infected animals. (**B**) Correlation of VLP-ELISA titers and neutralizing titers of all L1_LONG_-/L1_SHORT_-positive sera (correlation coefficient, R^2^ = 0.9883). The regression line represents a linear regression fit (Please note, that for both assays all sera were diluted in three-fold dilution steps. Since the titers are calculated from the dilution, data points of 294 different sera overlay when having the same titer in both assays). (**C**) Correlation of GST-L1_LONG_ ELISA and neutralization assay for 234 L1_LONG_-positive/L1_SHORT_-negative sera (R^2^ = 0.0000). Correlation analyses contain sera from animals representing the complete age range. Dashed lines indicate the methods’ cut-offs (OD_450_ = 0.2 for GST-ELISA or titer of 300 for neutralization assay).

Due to the different temporal order of seroconversion against L1_LONG_, L1_SHORT_ and VLPs, we reasoned that MnPV escapes from adaptive immunity to establish an efficient infection and to maintain a persistent life cycle, which is indicated by the increased seroresponse against MnPV E2 (Figure 2D). To get insight into this question, pseudovirion-based neutralization assays (PBNA) (Pastrana et al., 2004; Roden et al., 1996; Vinzón et al., 2014) were performed to monitor for the presence of protecting antibodies. As shown in Figure 4A, PBNA revealed a similar kinetics as previously demonstrated for the L1_SHORT_ isoform, indicating that neutralizing antibodies in fact appeared delayed. This was further substantiated by correlating the serum titers measured by VLP-ELISA with data obtained by PBNA (Figure 4B). Conversely, all sera directed against L1_LONG_ (but negative for L1_SHORT_) lack neutralizing capability (Figure 4C).

### Mapping of immunodominant epitopes in MnPV L1

**Figure 5.**
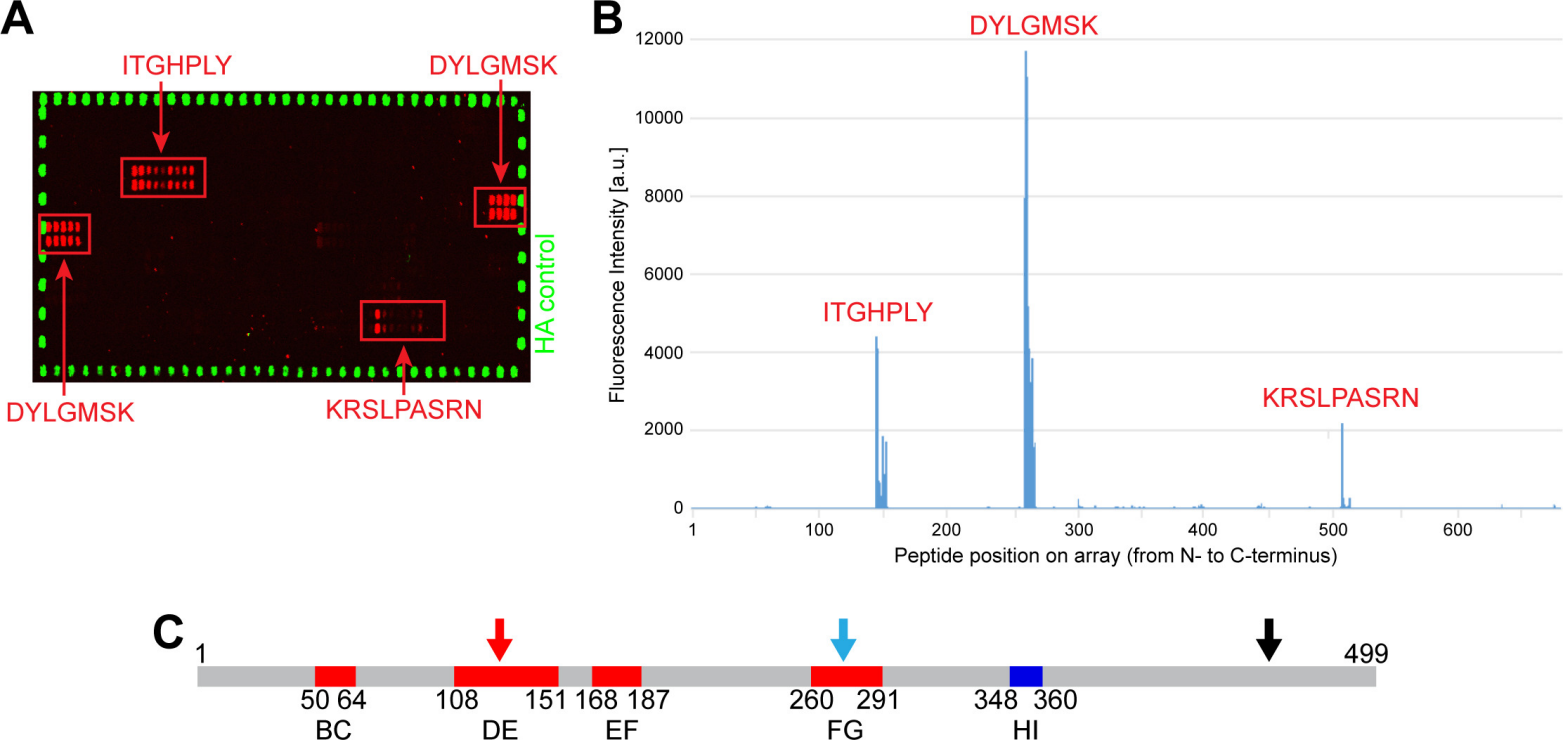
Peptide arrays identify known immunogenic epitopes in L1. (**A**) Synthetic 15-mer peptides with residue overlaps of 14 residues were spotted on microarrays and incubated with serum mix from five tumor-bearing animals with high titers against both L1 isoforms. Bound serum antibodies were detected with fluorophore-conjugated secondary antibodies. Positive regions (ITGHPLY, DYLGMSK and KRSLPASRN) are indicated and were mapped to their position in L1_LONG_ (**B**). (**C**) Two of these regions (ITGHPLY and DYLGMSK) coincide with the DE and the FG loop, respectively (scheme shows MnPV L1_SHORT_; see Supplementary file 1).

In order to identify epitopes within L1 recognized by the sera, synthetic linear 15-mer peptides with 14 residue overlaps were spotted on microarrays. Incubation with a *Mastomys* serum mix obtained from five tumor-bearing animals, possessing high titers against L1_LONG_ and L1_SHORT_ identified three immunogenic epitopes within the region homologous between L1_LONG_ and L1_SHORT_ (ITGHPLY, DYLGMSK and KRSLPASRN) (Figure 5A and B). Notably, two of them (ITGHPLY, DYLGMSK) coincide with the DE and FG loops (Figure 5C; Bissett et al., 2016) that form conformational epitopes on the surface of HPV virions (Li et al., 2017; Zhang et al., 2016) and are known to be highly immunogenic.

**Figure 5—figure supplement 1.**
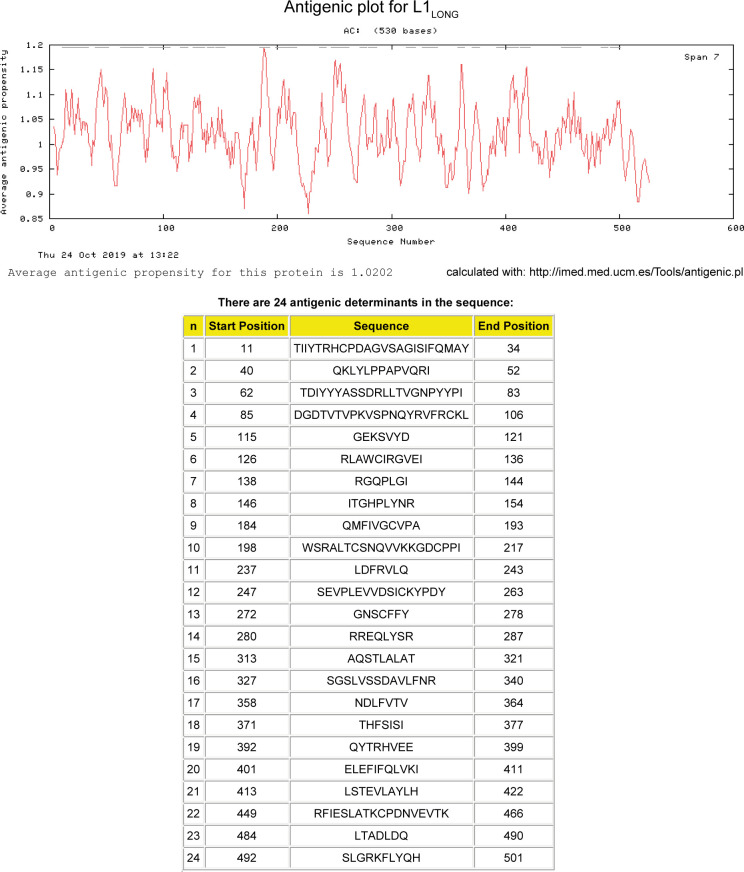
Immunogenicity prediction of L1_LONG_. Prediction of antigenic determinants within L1_LONG_ suggests an immunogenic epitope between residues 11 and 34 of the N-terminus of L1_LONG_. Also the DE loop (determinant no. 8) and partially the FG loop (last 2 residues of determinant no. 12) are predicted.

### Epitope characterization of anti-L1_LONG_ and anti-L1_SHORT_ antibodies

**Figure 6.**
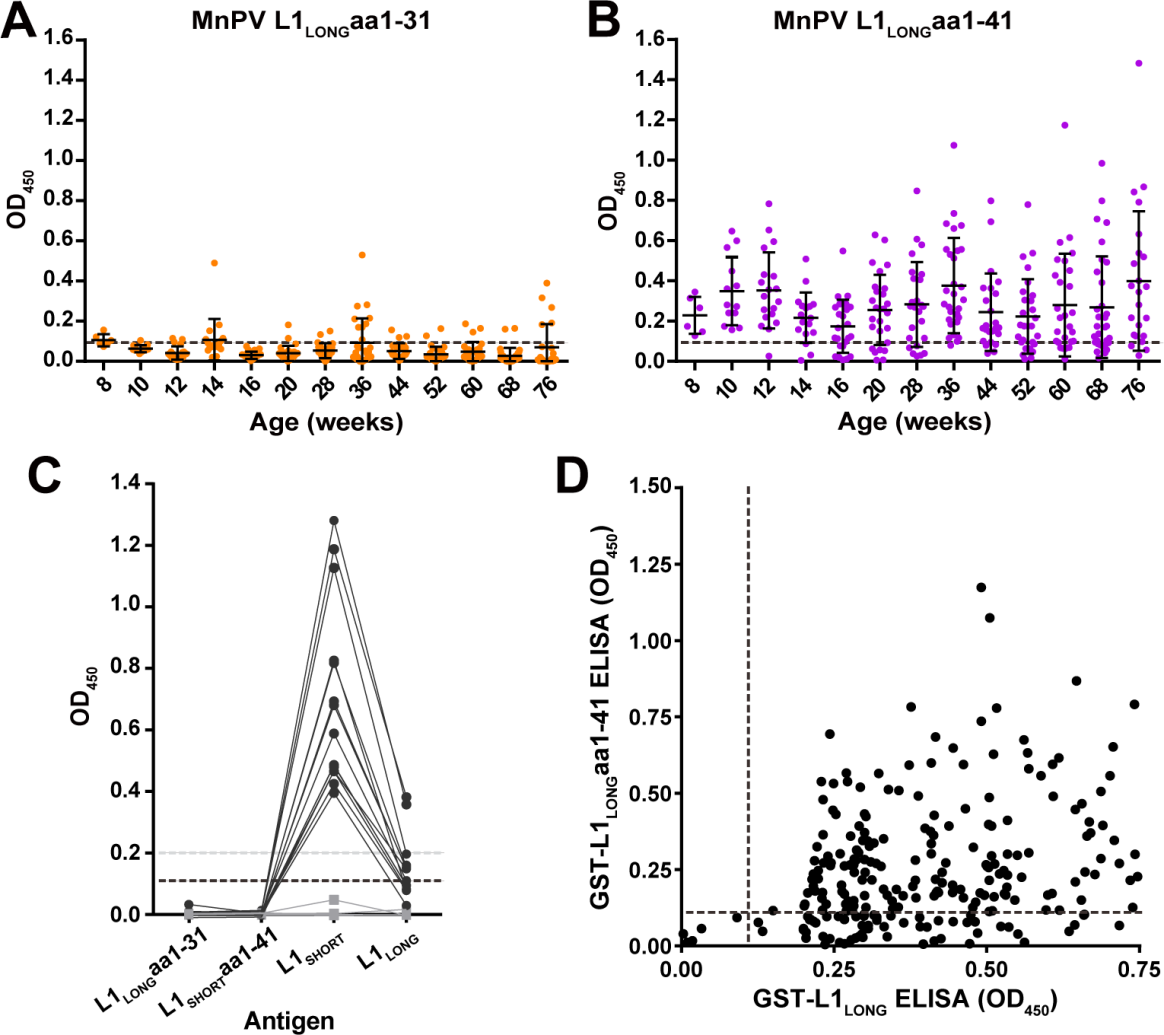
Seroreactivity against the N-terminus of L1_LONG_ measured by GST-ELISA. (**A**) Seroreactivity against the 31 aa exclusive for L1_LONG_ (60 animals, 682 sera) and (**B**) Against 41 aa of the N-terminus of L1_LONG_ (39 L1_LONG_-positive animals, 297 sera). Dashed lines represent the cut-off (0.11) based on virus-free animals. (**C**) Sera from MnPV-free animals (14 sera, black dots) vaccinated with VLPs (made from L1_SHORT_) and six pre-immune sera (grey squares) from a previous study (Vinzón et al., 2014) measured in the different L1 GST-ELISAs. Dashed lines indicate the cut-offs (grey: OD_450_ = 0.2 for L1_SHORT_ and L1_LONG_; black: OD_450_ = 0.11 for L1_LONG_aa1-31 and L1_LONG_aa1-41). (**D**) Correlation of ELISAs for GST-L1_LONG_ and GST-L1_LONG_aa1-41 (correlation coefficient, R^2^ = 0.0996).

To further elucidate why anti-L1_LONG_ antibodies lack neutralizing capacity, we dissected the seroresponse against L1 with respect to the 31 amino acids present only at the N-terminus of L1_LONG_ (Figure 1). Analyzing 297 sera of 39 L1_LONG_-positive animals by GST-ELISA, no detectable or only weak positivity could be measured against this L1_LONG_aa1-31 (Figure 6A). In contrast, when extending these 31 amino acids of L1_LONG_ to 41 residues (which includes nine residues of L1_SHORT_), the number of seropositive animals strikingly increased (Figure 6B), whereas such a reactivity could not be observed in sera from MnPV-free animals vaccinated with VLPs (made from L1_SHORT_) obtained in a previous study (Vinzón et al., 2014; Figure 6C). Correlating reactivities between L1_LONG_ and L1_LONG_aa1-41 (Figure 6D) shows that actually all sera which are positive for L1_LONG_aa1-41 are also positive for L1_LONG_, (which is not the case for L1_SHORT_, see Figure 6—figure supplement 1). It is therefore reasonable to assume that this antibody population is likely arising from exposure of the immune cells to the L1_LONG_ antigen.

**Figure 6—figure supplement 1.**
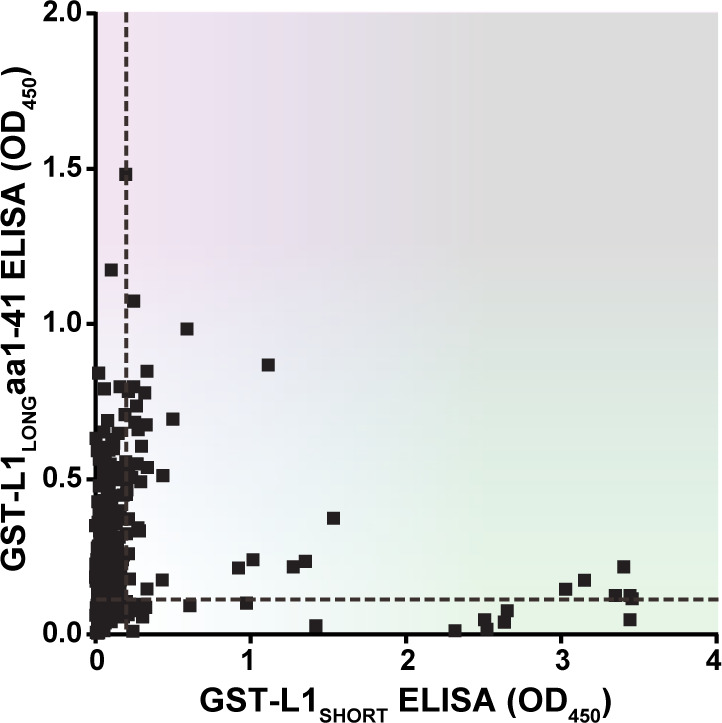
Additional correlation of seroreactivities measured by GST-ELISA. Correlation of GST-L1_SHORT_ with GST-L1_MIDDLE_ (R^2^ = 0.0106). Dashed lines indicate the cut-offs (OD_450_ = 0.2 for GST-L1_SHORT_, GST-L1_MIDDLE_ and GST-L1_LONG_; OD_450_ = 0.11 for L1_LONG_aa1-41).

These data, together with the finding that the first linear epitope recognized on the peptide array is located in L1_SHORT_ (Figure 5), indicate that a conformational epitope is spanning L1_LONG_ and L1_SHORT_, and represents both the most immunogenic epitope in L1_LONG_ and a major immunogen in early stages of infection. Moreover, an algorithm that predicts antigenic sites on proteins (Kolaskar and Tongaonkar, 1990), calculates the abovementioned DE loop (determinant no. 8) and partially the FG loop (determinant no. 12) (Figure 5—figure supplement 1) detected by the serum mix (see Figure 5) and also predicts an N-terminal epitope between residues 11 and 34 (determinant no. 1).

**Figure 7.**
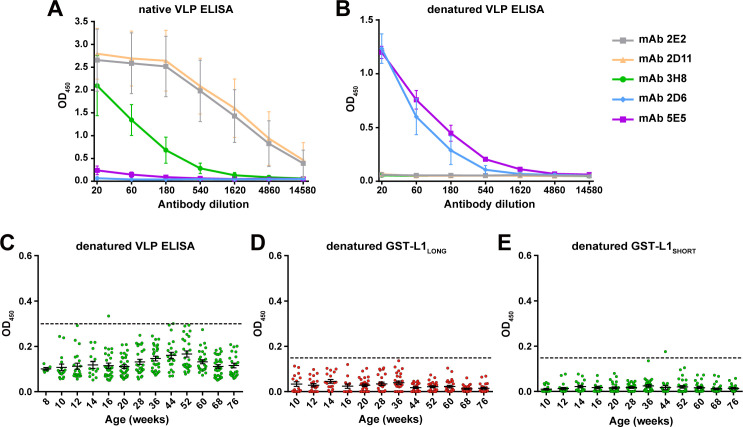
L1 denaturation abolishes recognition by naturally-raised antibodies. Binding efficacy of five monoclonal antibodies against (**A**) native VLPs and (**B**) VLPs denatured by heating (Mean ± SD, n = 3). (**C**) Seroresponse of *Mastomys* sera (306 sera positive for L1_LONG_ and L1_SHORT_) against denatured VLPs. (**D**) Seroresponse of *Mastomys* sera against denatured GST-L1_LONG_. (**E**) Seroresponse of *Mastomys* sera against denatured GST-L1_SHORT_. For (**E** and **D**), 281 sera from 40 animals positive for L1_LONG_ and L1_SHORT_ were measured. Dashed lines indicate the cut-offs (OD_450_ = 0.3 for denatured VLP-ELISA; OD_450_ = 0.15 for denatured GST-ELISA).

To further characterize the antigenic properties and to prove that a conformational epitope is formed at the N-terminus of L1_LONG_, ELISAs with denatured antigens (VLPs or GST fusion proteins) were performed. For this purpose, a panel of monoclonal antibodies raised against MnPV VLPs was used. These antibodies differ in terms of neutralization and sensitivity in VLP and GST-ELISAs (Supplementary file 2). Of these, only mAb 2E2, 2D11 and 3H8 showed high reactivity against native VLPs (Figure 7A, grey, yellow, and green lines), indicative for the recognition of conformational epitopes. Conversely, mAb 2D6 and 5E5 that possess low binding to intact VLPs were expected to represent antibodies recognizing linear epitopes (Figure 7A, blue and purple lines). To test this assumption, we anticipated a reversed pattern upon denaturation where the latter antibodies can bind, while binding of mAb 2E2, 2D11 and 3H8 should be abrogated. As shown in Figure 7B, this was indeed the case (see also Supplementary file 2).

Sera from the collective of 60 naturally infected animals previously tested positive for L1_LONG_ and L1_SHORT_ were also tested in denatured-VLP ELISA (306 sera) and denatured-GST-L1 ELISA (281 sera). Interestingly, denaturation of both VLPs and GST-L1 antigens abrogated the reactivity of all sera (Figure 7C–E), suggesting that anti-L1_LONG_ and anti-L1_SHORT_ antibodies were indeed directed against conformational epitopes.

### L1_SHORT_ but not L1_LONG_ and L1_MIDDLE_ can form VLPs and infectious pseudovirions

**Figure 8.**
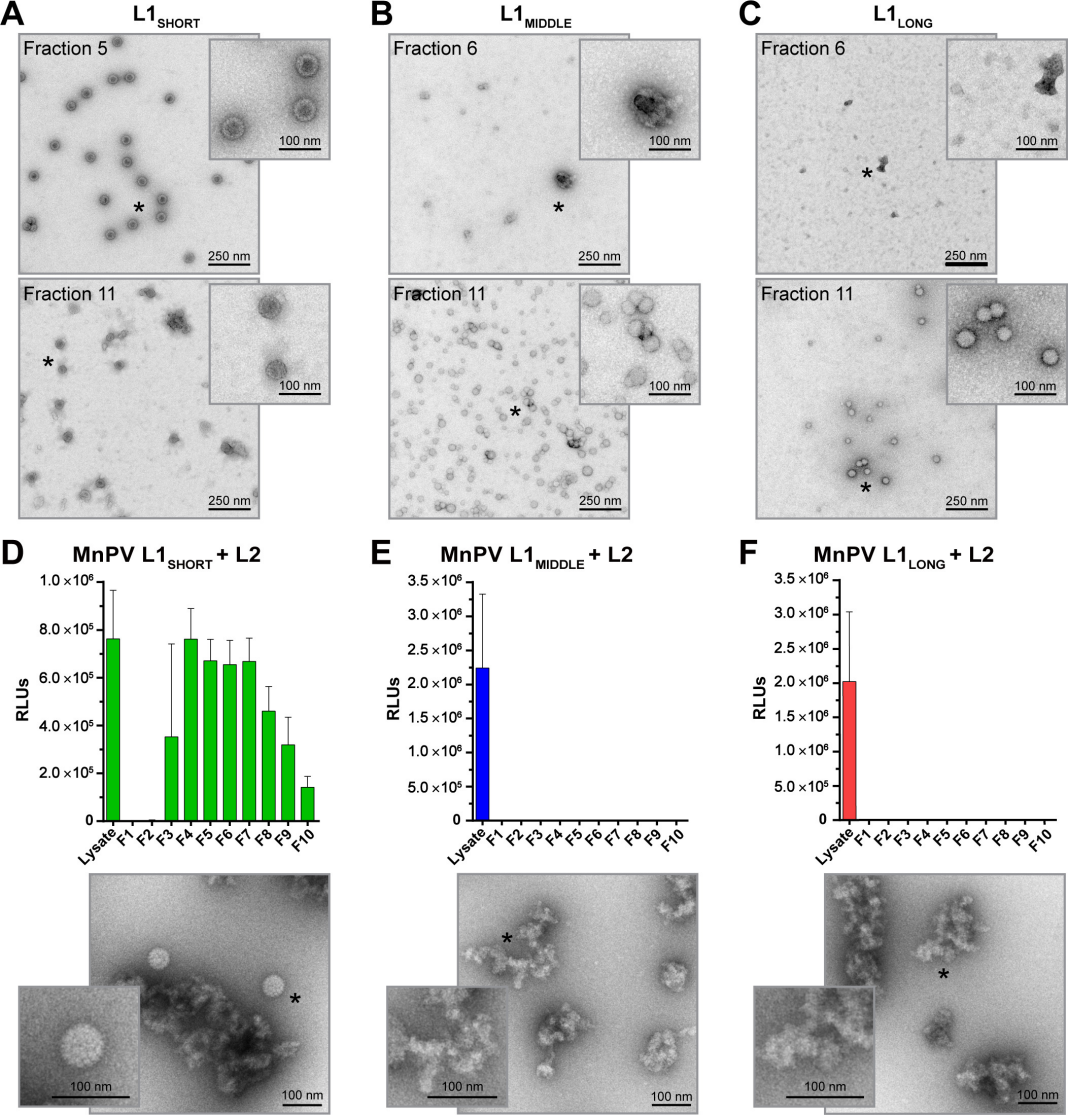
VLP and pseudovirion formation capacity of L1_SHORT_, L1_MIDDLE_ and L1_LONG_. EM micrographs of peak fractions and the respective fractions of lowest densities of (**A**) L1_SHORT_ (**B**) L1_MIDDLE_ and (**C**) L1_LONG_ at 16,000x magnification. Capacity to form infectious pseudovirions in presence of L2 was analyzed by infectivity assay and EM for (**D**) L1_SHORT_ (**E**) L1_MIDDLE_ and (**F**) L1_LONG_. Note that high signals with unpurified lysate from PsV-producing cells result from co-expressed luciferase reporter protein (Mean ± SD, n = 3).

To test the different isoforms for their capability to form virus-like structures, the ORFs of L1_SHORT_, L1_MIDDLE_ and L1_LONG_ were expressed in Sf9 insect cells by the use of recombinant baculoviruses. Different preparations were analyzed by CsCl density gradient centrifugation. For quality control, the gradients’ refractive indices were measured and corresponding fractions were analyzed via western blot, where all three L1 isoforms could be found, ranging between 55 and 70 kDa (Figure 8—figure supplement 1). To analyze the ability of the isoforms to form VLPs or similar structures, different fractions of the gradients were examined by EM. Considering L1_SHORT_, highly concentrated and spherically shaped particles with sizes of 60 nm and clearly visible capsomers could be found (Figure 8A). Also in the lowest density fractions (e.g. fraction 11), particles with capsomer-like structures were detected. Conversely, inspecting the gradients of L1_LONG_ and L1_MIDDLE_, similar assemblies were absent, although many particles of different sizes (20–50 nm) were found (Figure 8B and C). However, it is difficult to classify these substructures as regular capsomers, indicating that L1_LONG_ and L1_MIDDLE_ were apparently not able to form correctly assembled VLPs under the same experimental conditions.

**Figure 8—figure supplement 1.**
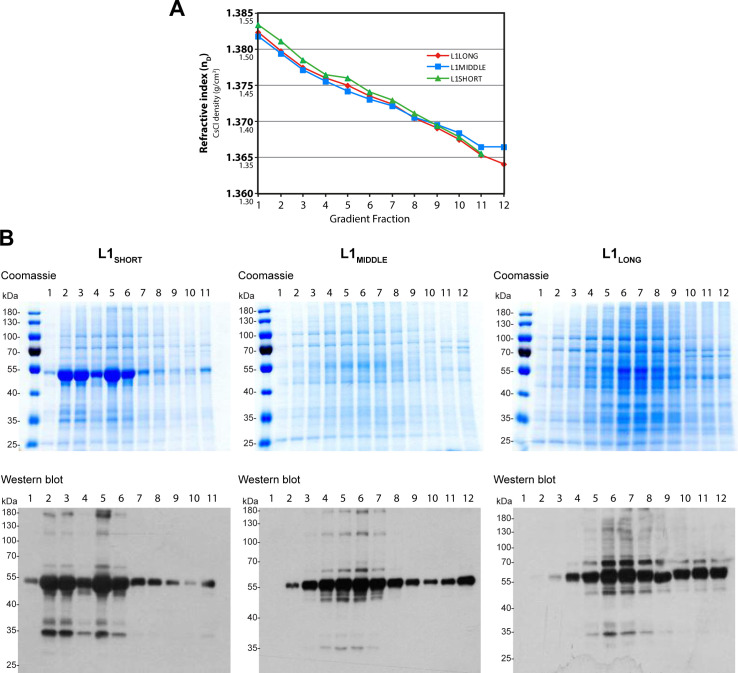
Production of L1 isoforms with the MultiBac baculovirus expression system. (**A**) The CsCl density (refractive index) of each fraction was determined via refractometer at RT. (**B**) Analysis of fractions from PsV production with L1_SHORT_, L1_MIDDLE_ and L1_LONG_ analyzed by Coomassie blue staining (upper panels) and Western blot (lower panels). Immunoblots were incubated with a serum mix from five MnPV-infected tumor-bearing animals.

**Figure 8—figure supplement 2.**
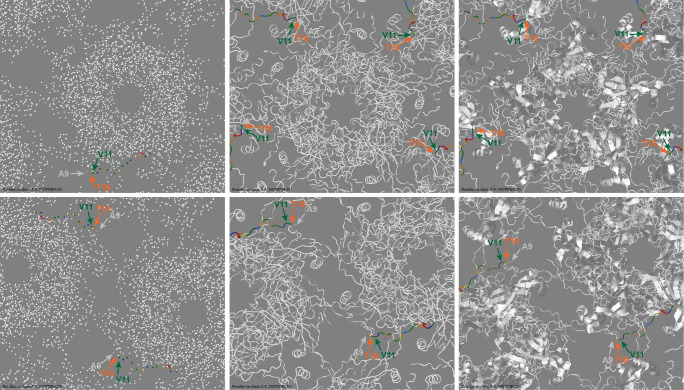
Pseudoatomic modeling of L1 proteins in the viral capsid. Model of HPV16 capsid (cryo-electron microscopy, structure ID: 3J6R, view from inside of the capsid, display: left: ball and stick, middle: trace, right: cartoon). The chain starts at residue 9. The first three residues ATV of the different L1 proteins are indicated.

To examine whether the addition of L2, regularly present in mature infectious virions, can facilitate the formation of L1_LONG_ or L1_MIDDLE_ composed particles, pseudovirions were produced. 293TT cells were transfected with expression plasmids encoding L2 and the different L1 isoforms in conjunction with a reporter plasmid. The assembled structures were purified via Optiprep gradients and infection assays with different fractions were performed. In contrast to L1_SHORT_ (Figure 8D), L1_LONG_ and L1_MIDDLE_ again did not yield virus-like structures even in the presence of L2 (Figure 8E and F). Moreover, while infectious L1_SHORT_-based pseudovirions can be found in most of the gradient fractions, no signals of the reporter construct could be discerned for L1_LONG_ and L1_MIDDLE_. Luciferase signals could be measured in non-fractionated 293TT cell lysates, indicating that the transfection was successful for all three L1 isotypes.

### L1_SHORT_ but not L1_LONG_ can form high-MW structures in genuine host cells

**Figure 9.**
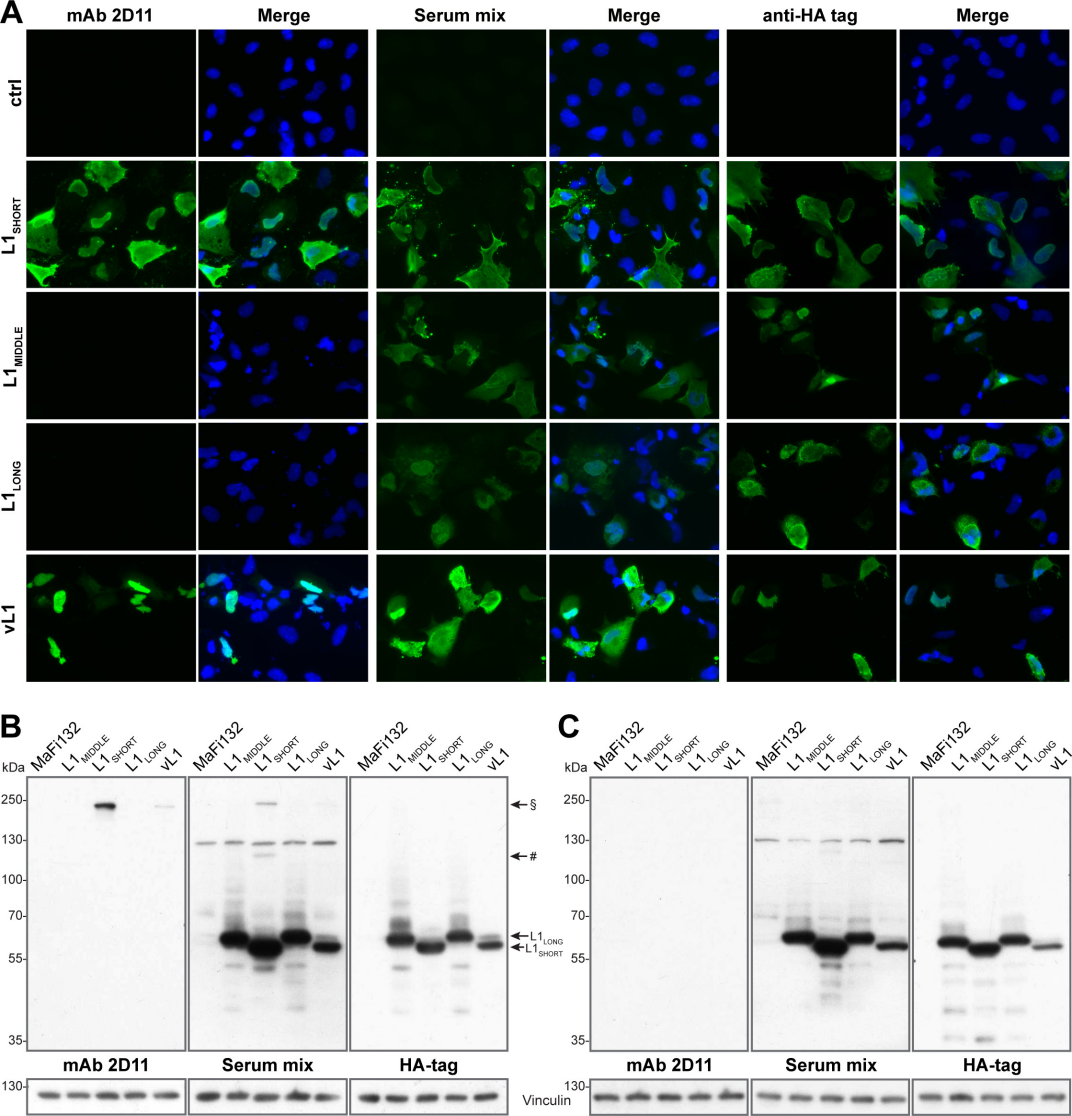
L1_SHORT_ and L1_LONG_ protein expose different epitopes when expressed in *Mastomys* cells. *Mastomys*-derived fibroblasts were transfected with HA-tagged L1_SHORT_, L1_MIDDLE_ or L1_LONG_ (humanized codons and artificial Kozak sequences), or the polycistronic plasmid vL1 (encodes all three viral L1 ORFs and Kozak sequences). (**A**) Expression of L1 isoforms was visualized with mAb 2D11 (only recognizing conformational L1 epitopes), serum mix from five tumor-bearing animals and anti-HA as a control. (**B**) Western blotting reveals two high-MW bands only in L1_SHORT_-expressing cells corresponding to L1 dimers (#) and trimers (§). Transfection with vL1 results in synthesis of both L1_LONG_ as well as L1_SHORT_ and its multimer. (**C**) Upon harsher denaturation of cell lysates, L1_SHORT_ multimer bands disappear. Vinculin served as loading control.

To further investigate L1 isoform expression in their genuine host in vitro, *Mastomys*-derived fibroblasts (Hasche et al., 2016) were transfected either with plasmids exclusively encoding HA-tagged L1_SHORT_, L1_MIDDLE_ or L1_LONG_ or with the polycistronic plasmid vL1 encoding the genuine viral L1 ORFs as found in natural transcripts. While serum mix from tumor-bearing animals was able to detect all L1 isoforms in immunofluorescence stainings, mAb 2D11 (which exclusively binds a conformational L1_SHORT_ epitope, see Figure 7D and E, respectively) could only detect L1_SHORT_ but not L1_MIDDLE_ and L1_LONG_, despite similar expression levels of all isoforms (see HA-tag) (Figure 9A). This indicates that in *Mastomys* cells only the L1_SHORT_ can form structures involved in the formation of virus particles.

Western blot analysis of the corresponding cells showed similar expression of all HA-tagged L1 isoforms, which again could be detected by the serum mix (Figure 9B). Notably, only in L1_SHORT_-transfected cells both mAb 2D11 and serum mix detected bands between 100 and 130 kDa and around 250 kDa, which disappeared in lysates treated with additional DTT, β-mercaptoethanol and extended heating prior to SDS-PAGE separation (Figure 9C). Their sizes correlate to L1-dimers and trimers and since mAb 2D11 does not detect linear epitopes, this suggests that only the L1_SHORT_ isoform is able to form capsomer-like structures, which are at least partially structured in non-reducing conditions due to stabilizing properties of inter-capsomeric disulfide bridges (Buck et al., 2005b; Sapp et al., 1998).

Previous viral transcriptome analysis revealed the presence of three polycistronic transcripts (referred to as Q, R and S) that have the potential to encode both L1_LONG_ and L1_SHORT_ (Salvermoser et al., 2016). Using the most abundant of these (transcript Q) for prediction of the start codons’ likelihood of being used for translation initiation (Nishikawa et al., 2000), it turned out that the ORFs of E1^E4 (reliability index, RI = 0.43), L2 (RI = 0.42) and L1_LONG_ (RI = 0.38) could be favored over L1_SHORT_ (RI = 0.17) (Supplementary file 3). Indeed, consistent with the immunofluorescence, when transfecting cells with the polycistronic construct vL1, L1_LONG_ as well as L1_SHORT_ and its multimer band are detected, which confirms that both ORFs are functional and that L1_LONG_, although unable to form capsomers, can be expressed from such a polycistronic construct (Figure 9).

### L1_LONG_ and L1_SHORT_ appear at different locations in MnPV-induced tumors

**Figure 10.**
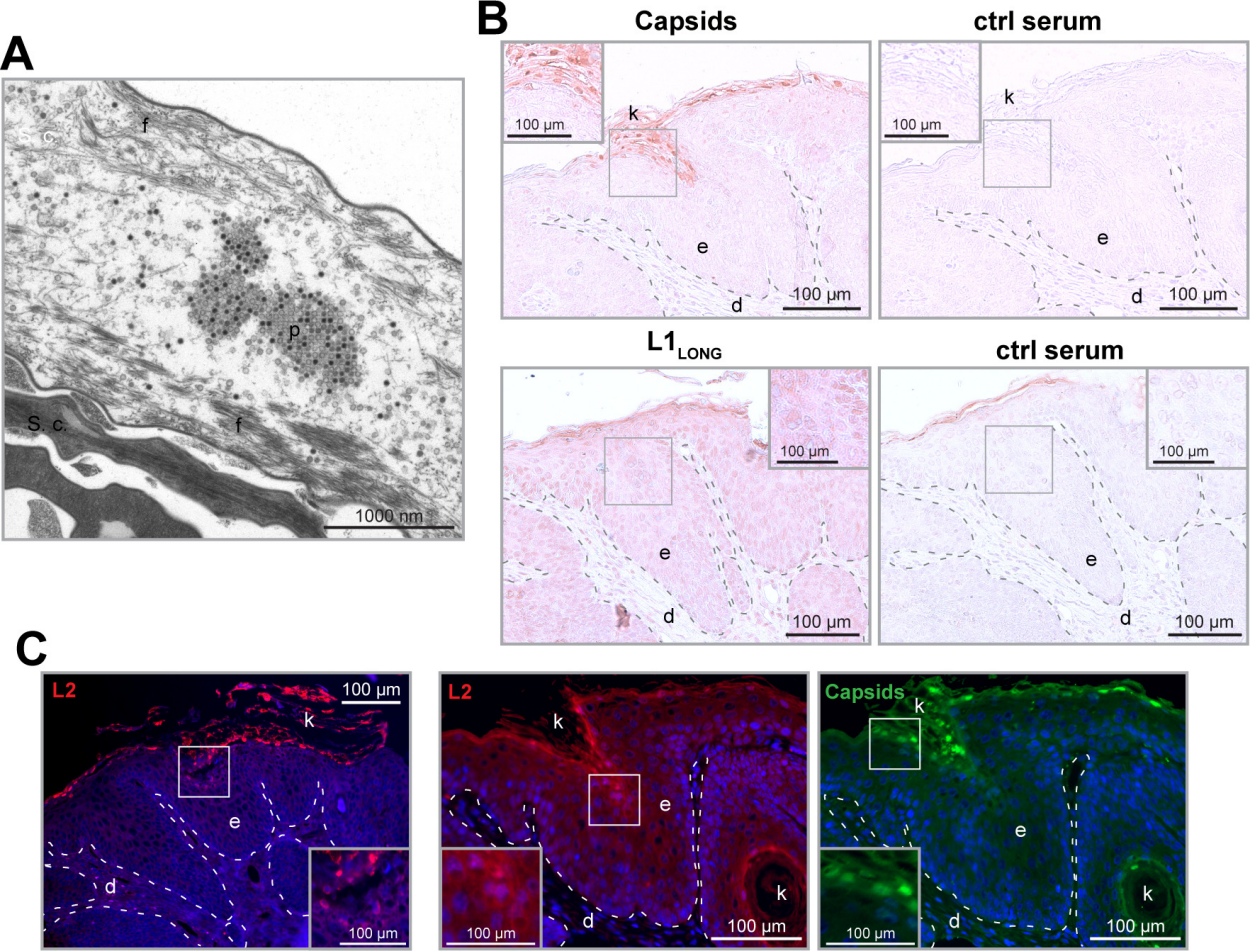
L1_LONG_ synthesis occurs much earlier than capsid formation in vivo. (**A**) EM micrograph of MnPV particles (p) in the *stratum corneum* (s.c.) of a MnPV-induced papilloma. The nearly shed cell shown here is strongly degraded and only tonofilaments (f) are left. (**B**) Immunohistochemical analysis reveals MnPV capsids (detected with serum of a MnPV-VLP-immunized *Mastomys*) in the uppermost layers of the *stratum corneum* while L1_LONG_ (detected by serum from a mouse immunized with the N-terminal 31 residues of MnPV-L1_LONG_) appears throughout the whole epidermis. Pre-immune sera were used as controls. (**C**) Tissue sections were stained with cross-reactive anti-HPV-L2 antibody (K18L2, red) or cross-reactive anti-HPV-VLP guinea pig serum (green) as controls. Consistent with capsid formation, L2 only appears in the uppermost layers of the tissue shortly prior to VLP formation (d: dermis, e: epidermis, k: keratin).

Considering the PV life cycle, virions are released by shedding of terminally differentiated cells at the uppermost layer (*stratum corneum*) of the epidermis. Here, L1 and L2 are assembled to capsomers and virus particles (Figure 10A). When staining MnPV-induced papillomas with serum that detects mature MnPV virions, virus particles can only be found in cornified structures above or within the epidermis and in islands of terminally differentiated keratinocytes (Figure 10B). This is consistent with the expression of L2, which also appears the earliest in nearly shed cells (Figure 10C). Conversely, using serum from mice immunized with the N-terminal peptide exclusive for L1_LONG_, keratinocytes in the basal layer and the complete epithelium are positively stained (Figure 10B). This suggests that its expression takes place already during early infection phases long before viral particles are formed.

## Discussion

The non-random distribution of several ATG initiation codons within the L1 open reading frame (ORF) of certain human and animal papillomaviruses (Figure 1) potentially allows the translation of different L1 isoforms. Despite plenty of seroepidemiological studies on HPV, to our knowledge, seroresponses against different L1 isoforms have never been performed. Previous studies only focused on the shortest variant, known to efficiently form virus-like particles (VLPs) or, together with L2, infectious virions (Buck et al., 2005b).

The MnPV-infected rodent *Mastomys coucha* is a reliable preclinical model that mimics many aspects of the situation found in humans infected with cutaneous HPVs. Since the animals are immunocompetent and become naturally infected early in life (Hasche and Rösl, 2019; Hasche et al., 2018), they represent a unique virus-host system to investigate the humoral response against MnPV L1 isoforms during the natural course of infection.

Of note was the finding that seroconversion against the isoforms L1_LONG_ and L1_MIDDLE_ appeared strikingly earlier than for L1_SHORT_ (Figure 2), raising the question of the selective advantage for the virus and in turn its permissive cycle. To address this issue, pseudovirion-based infection assays were performed. Here, only sera binding L1_SHORT_ inhibited pseudovirion infection, while sera recognizing the L1_LONG_ and L1_MIDDLE_ isoforms were not neutralizing (Figure 4). Furthermore, in contrast to L1_SHORT_, the longer isoforms were also not able to form intact viral particles (Figure 8).

Conversely, the time course of MnPV E2 seroreactivity (Figure 2D) in comparison to the appearance of neutralizing antibodies (Figure 4) is indicative for viral replication and spread of infection (Schäfer et al., 2011; Xue et al., 2010). It is therefore reasonable to assume that the delay of neutralization capacity apparently allows the virus to accumulate. Pointing in this direction is also the kinetics of seroconversion against the L2 minor capsid protein (Figure 2E), finally assembling with L1 to form new infectious progeny virions at the end of the permissive cycle. Moreover, the correlation of the different ELISAs showed an obvious consistency between VLP and GST-L1_SHORT_ that is not maintained when VLP or GST-L1_SHORT_ are compared with GST-L1_LONG_ (Figure 3).

As reported from mice immunized with recombinant HPV VLPs, neutralizing antibodies act via binding to linear or conformational epitopes (Combita et al., 2002; Senger et al., 2009a; Zhang et al., 2016). Although most high-affinity neutralizing antibodies bind to conformation-dependent epitopes, antibodies recognizing linear epitopes can be raised against improperly maturated VLPs (used for immunization) or virions (in the course of a natural infection) (Senger et al., 2009a). Similar to HPV16 L1 (Bissett et al., 2016; Zhang et al., 2016), immunodominant epitopes could be identified within the DE and FG loops of MnPV L1 (Figure 5), known to form highly immunogenic conformational epitopes on the surface of virions (Li et al., 2017; Zhang et al., 2016).

Interestingly, we identified a novel epitope in the N-terminal region of L1, spanning at least some of the residues exclusive for L1_LONG_ and extending to the first amino acids from L1_SHORT_. Seroconversion against this peptide (referred to as L1_LONG_aa1-41 in Figure 6B) followed roughly the same kinetics as L1_LONG_ (Figure 2B) and most L1_LONG_-positive sera recognized these 41 residues (Figure 6D). However, all sera only positive for L1_LONG_, but negative for L1_SHORT_ did not neutralize PsVs (Figure 4C) and further lost their ability to bind to L1_LONG_ when the antigen was denatured prior to the ELISA (Figure 7D). Hence, the addition of N-terminal residues to L1_SHORT_, obviously leads to a distinct folding of L1_LONG_, thereby forming a new conformational epitope, which is not present in L1_SHORT_-derived VLPs and natural virions that do not induce such antibodies upon immunization or infection (Figure 6C). Due to the fast kinetics of seroconversion against L1_LONG_, this conformational epitope is apparently the predominant immunogenic structure in L1_LONG_ and most probably masks neutralization epitopes that would be accessible in VLPs (Figures 2F and 4A).

Indeed, in the case of HPV16 L1, the length of the N-terminus is decisive for efficient and correct assembly of L1 into VLPs (Chen et al., 2000). Consistently, neither MnPV L1_LONG_ nor L1_MIDDLE_ formed particles in the right size and shape in insect cells (Figure 8), a finding also reported for the mouse papillomavirus MmuPV L1 (Joh et al., 2014). Moreover, while co-expression of MnPV L1_SHORT_ and L2 results in well-shaped infective pseudovirions, L1_LONG_ and L1_MIDDLE_ strikingly inhibited pseudovirion formation (Figure 8), suggesting that the N-terminus is literally hindering the assembly of both isoforms to capsids. Previously, crystallization of HPV L1 for 3D structure determination was not successful with the ‘usual’ L1 (being L1_SHORT_) but required its N-terminal truncation (Chen et al., 2000), probably because the crystallization conditions lead to a folding of the N-terminus that prevents particle formation. Therefore, even the most complete 3D model 3J6R for HPV16 L1 (Cardone et al., 2014) lacks eight N-terminal residues. It was speculated that the N-terminus of L1 fills a gap in interpentameric structures (Figure 8—figure supplement 2) and since N-terminal deletion of eight residues has such a big impact on particle formation (Chen et al., 2000) it is likely that the additional 31 residues do not fit into this gap and furthermore completely sterically hinder L1 assembly. Additionally, the extended N-terminus not only inhibits assembly, but also causes a complete new folding of L1, thereby exposing a novel immunogenic conformational epitope that induces non-protective antibodies.

Nonetheless, using *Mastomys*-derived cells as genuine in vitro system to monitor expression of the three MnPV L1 variants (Figure 9), we found that L1_SHORT_ but not the larger isoforms are recognized by mAb 2D11, confirming the presence of conformational epitopes found in capsomers. Importantly, this experiment also showed that L1_LONG_ and L1_SHORT_ ORFs are functional in a polycistronic setting and can be translated in *Mastomys* cells.

PV late gene expression is tightly regulated at transcriptional and post-transcriptional level (Graham, 2017). The MnPV transcription map obtained from skin lesions revealed L1 transcripts to be the most abundant in productive infections, which are encoded by polycistronic mRNAs (coding for E1^E4, L2 and L1) exclusively controlled by the late viral promoter (Salvermoser et al., 2016). However, speculating about the mode of L1 isoform regulation, the following scenario could be envisioned: late PV transcripts are known to be strongly upregulated upon keratinocyte differentiation. However, due to insufficient suppressed late viral promoter activity, late transcripts can already be detected in undifferentiated cells (Songock et al., 2017). Indeed, in contrast to mucosal types (e.g. HPV11, 16 and COPV), cutaneous papillomaviruses (e.g. HPV1, 2 and BPV1) initiate their late functions in the lower epithelial regions (Peh et al., 2002). Consequently, this could lead to synthesis of the highly immunogenic L1_SHORT_ already in early stages of infection, giving rise to neutralizing antibodies, thereby counteracting the permissive cycle and in turn the viral spread. However, the predicted presence of a stronger Kozak sequence in a upstream ORF from the conventional L1 (L1_SHORT_) (Supplementary file 3), generates an alternative longer isoform of L1 which is expressed already in basal and suprabasal layers of MnPV-induced lesions (Figure 10) and similar to the pattern of L1 mRNA expression in MmuPV1-induced papillomas (Xue et al., 2017). This L1_LONG_ isoform is also immunogenic but does not raise neutralizing antibodies.

Conversely and in agreement with other studies (Biryukov and Meyers, 2015; Borgogna et al., 2014), capsid formation could only be found in the granular and uppermost layers (Figure 10), which are less accessible for immune effector cells. However, based on in silico prediction, the capsid-forming L1_SHORT_ cannot be efficiently translated from polycistronic transcripts coding for E1^E4, L2 and L1 (Supplementary file 3), reinforcing the existence of a transcript in which the splicing acceptor is located immediately upstream of the L1_SHORT_ AUG (Salvermoser et al., 2016). This splicing event is more tightly regulated than late promoter activity, based on a splicing silencer sequence suppressing the use of this site during mRNA processing (Zhao et al., 2004). Thus, only the combination of splicing regulation and the presence of favored ORFs ensure L1_SHORT_ synthesis in upper layers of the epithelium (Zhao et al., 2004). This may allow a better escape from immune surveillance until the regular permissive cycle is completed.

Finally, the question remains why only certain PVs produce an immunogenic L1 isoform that is not needed for its own life cycle. Considering a virus-host interaction as consequence of an evolutionary process, there is a sophisticated balance between host immune surveillance and immune escape by the respective virus to ensure virus progeny production (French and Holmes, 2020). The temporal and spatial change of such an equilibrium in favor to infection determines the efficiency of viral accumulation and maturation as well as the spread to infect another host (Rothenburg and Brennan, 2020).

The occurrence of several L1 ORFs is not a peculiarity of MnPV, but also found in different HPV genera as well as in animal papillomaviruses such as MmuPV (Joh et al., 2014; Webb et al., 2005). It is intriguing that alternative ORFs are mostly present in mucosal ‘high-risk’ HPV types that cause clinical symptoms, but not in ‘low-risk’ types such as HPV6 and 11 (Webb et al., 2005). Hence, there must be a selection pressure to maintain several L1 initiation codons in these PVs types, thereby allowing the synthesis of different isoforms to get an advantage for the virus.

Naturally occurring mutations within neutralizing epitopes that reduce the antigenicity of HPV L1 and L2 proteins may contribute to humoral immune evasion (Seitz et al., 2013; Yang et al., 2005). Such L1 variants, isolated from premalignant HPV16 positive cervical tissue, showed impaired viral capsid assembly that can influence both B cell class switching and the production of non-neutralizing antibodies (Yang et al., 2005). Indeed, assembly-defective HPV16 VLPs impair the activation of dendritic cells that play a decisive role in activating adaptive immunity (Yang et al., 2005). Whether this also explains the late development of HPV16 L1 neutralizing antibodies (eight and nine months after the first positive HPV DNA detection) (Gutierrez-Xicotencatl et al., 2016) remains to be elucidated.

In conclusion, it is tempting to speculate that our results show that early synthesis of alternative immunogenic L1 isoforms represents a novel mode of humoral immune escape mechanism, favoring persistent infections and viral spread due to a delay of immune recognition by the host.

## Materials and methods

**Key resources table:** 

Reagent type (species) or resource	Designation	Source or reference	Identifiers	Additional information
Genetic reagent (*Mastomys coucha*)	African Multimammate rodent	DKFZ, Prof. F. Rösl	*Mastomys coucha*	Used asexperimental model, Hasche and Rösl, 2019
Gene (*Mastomys natalensis Papillomavirus1*)	MnPV	GenBank	NC_001605.1	Tan et al., 1994
Biological sample (*Mastomys coucha*)	Sera	This paper		Sera tested for seroconversionagainst different MnPV proteins
Biological sample (*Mastomys coucha*)	Sera	Vinzón et al., 2014		Sera from VLP-vaccinated animals
Cell line (*Mastomys coucha*)	MaFi132; *Mastomys coucha-* derived fibroblasts	DKFZ, Prof. F. Rösl		Hasche et al., 2016
Cell line (*Homo sapiens*)	HeLaT	DKFZ, Prof. M. Müller, Sehr et al., 2002	HeLaT clone-4,	Used for pseudovirion--based neutralization assay
Cell line (*Homo sapiens*)	293TT	DTP, DCTD TUMOR REPOSITORY	NCI-293TT; RRID:CVCL_1D85	Used forpseudovirion production
Cell line (*Spodoptera frugiperda*)	Sf9	DKFZ, Prof. M. Müller	RRID:CVCL_0549	Insect cells,used for VLPproduction
Cell line (*Trichoplusia ni*)	TN-High Five	Gibco	BTI-TN-5B1-4; RRID:CVCL_C190	Insect cells, used for VLPproduction
Strain, strain background (*Escherichia coli*)	TOP10 (DH10B)	Invitrogen	Cat#: C404010	Chemically competent cells
Strain, strain background (*Escherichia coli*)	DH10MultiBac^Cre^	Geneva Biotech	Electrocompetent cells	Fitzgerald et al., 2006
Transfected construct (*Mastomys natalensis Papillomavirus1*)	pFBDM_L1_SHORT_, pFBDM_L1_MIDDLE_ , pFBDM_L1_LONG_	This paper	Backbone RRID:Addgene_110738	Multibac constructs to transfect andexpress MnPVL1 variants ininsect cells for VLPproduction
Transfected construct (*Mastomys natalensis Papillomavirus1*)	pPK-CMV-E3_L1_SHORT_, pPK-CMV-E3_L1_MIDDLE_, pPK-CMV-E3_L1_LONG_	This paper		Constructs totransfect humanized ORFs andexpress MnPVL1 variants inMaFi132 cells
Transfected construct (*Mastomys natalensis Papillomavirus1*)	pPK-CMV-E3_vL1	This paper		Construct totransfect and express all L1 ORFs as found in the genuineMnPV genomein MaFi132 cells
Biological sample (*Mastomys natalensis Papillomavirus1*)	MnPV VLPs	This paper		MnPV virus-likeparticles usedfor VLP-ELISAand assemblystudies
Biological sample (*Mastomys natalensis Papillomavirus1*)	MnPV PsVs	This paper		MnPV pseudovirions for infectivity assay andPBNA
Antibody	anti-L1 (Mouse monoclonal)	This paper	mAb 2E2, 2D11, 3H8, 2D6, 5E5	ELISA (1:20-1:14,580), IF(1:5), WB(2D11, 1:1000)
Antibody	*Mastomys* serum mix (*Mastomys coucha* polyclonal serum)	This paper		IF (1:1000),Peptide Array(1:300)
Antibody	Anti-HA clone 3F10 (Rat monoclonal)	Sigma-Aldrich	Cat#: 11867423001; RRID:AB_390918	IF (1:1000), WB (1:1000)
Antibody	Anti-Vinculin clone 7F9 (Mouse monoclonal)	Santa Cruz	Cat#: sc-73614; RRID:AB_1131294	WB (1:4000)
Antibody	Anti-L1_LONG_aa1-31 serum (Mouse polyclonal serum)	This paper		IHC (1:100)
Antibody	Anti-L2 serum (Guinea pig polyclonal serum)	DKFZ, Prof. M. Müller		IHC (1:200)
Antibody	Anti-L2 clone K18L2 (Guinea pig polyclonal serum)	DKFZ, Prof. M. Müller, Rubio et al., 2011		IHC (1:200)
Antibody	Anti-Mouse IgG (H+L), HRP Conjugate (Goat polyclonal)	Promega	Cat#: W4021; RRID:AB_430834	ELISA (1:10,000), WB (1:10,000)
Antibody	Peroxidase AffiniPure Goat Anti-Rat IgG (H+L)	Jackson ImmunoResearch	Cat#: 112-035-003; RRID:AB_2338128	WB (1:10,000)
Antibody	Goat anti-Mouse IgG (H+L), Alexa Fluor 488 (Goat polyclonal)	Invitrogen	Cat#: A11029; RRID:AB_138404	IF (1:1000), IHC (1:1000)
Antibody	Goat anti-Guinea Pig IgG (H+L), Alexa Fluor 488 (Goat polyclonal)	Invitrogen	Cat#: A11073; RRID:AB_2534117	IHC (1:1000)
Antibody	Donkey anti-Rat IgG (H+L), Alexa Fluor 488, (Donkey polyclonal)	Invitrogen	Cat#: A21208; RRID:AB_141709	IF (1:1000)
Antibody	Mouse IgG (H and L) Antibody DyLight 680 Conjugated	Rockland Immunochemicals	Cat#: 610-144-121; RRID:AB_1057546	Peptide Array (1:5000)
Recombinant DNA reagent	pPK-CMV-E3	Promocell	Cat#: PK-MB-P003300	
Peptide, recombinant protein	GST-L1_SHORT_, GST-L1_MIDDLE_, GST-L1_LONG_,	This paper, Schäfer et al., 2010		GST proteinfused to thedifferent MnPV L1 variants
Peptide, recombinant protein	GST-L1_LONG_aa1-31, GST-L1_LONG_aa1-41	This paper		GST proteinfused to the N-terminus of MnPV L1
Peptide, recombinant protein	GST-E2, GST-L2	This paper, Schäfer et al., 2010		GST proteinfused to MnPVE2 or L2
Peptide, recombinant protein	L1 peptide array	PEPperPRINT GmbH		Stadler et al., 2008
Commercial assay or kit	Dako REAL Detection System, Peroxidase/AEC, Rabbit/Mouse	Agilent	Cat#: K5007	IHC chromogenicdetection Kit
Commercial assay or kit	Gaussia glow juice	PJK Biotech	Cat#: 102542	Luciferase activity detection kit
Chemical compound, drug	DAPI	Sigma-Aldrich	Cat#: D9542-5MG	
Software, algorithm	GraphPad Prism 6.0	GraphPad		

### Animals

The *Mastomys coucha* breeding colony naturally infected by MnPV is maintained under SFP conditions in individually ventilated cages (Tecniplast GR900) at 22+/- 2°C and 55+/- 10% relative humidity in a light/dark cycle of 14/10 hr. *Mastomys* were fed with mouse breeding diet and allowed access to water ad libitum. For the follow-up experiment, animals were monitored for the duration of their lifetime until they had to be sacrificed due to tumor development or decrepitude. Blood was taken in intervals from 2–8 weeks by puncturing the submandibular vein of anesthetized animals (3% isoflurane), starting at the age of eight weeks.

### Cell culture conditions

293TT, HeLaT and MaFi132 cells were grown in DMEM supplemented with 10% fetal calf serum (FCS), 1% Penicillin/Streptomycin and 1% L-glutamine. Media of HeLaT and 293TT cell were further supplemented with Hygromycin B (125 μg/ml) to maintain additional SV40 large T-antigen expression. All cell lines were kept at 37°C, 5% CO_2_ and 95% humidity and regularly checked for Mycoplasma via PCR. Sf9 and TN-High Five insect cells were kept as described elsewhere (Senger et al., 2009b).

### GST-capture ELISA

As previously described (Schäfer et al., 2011; Schäfer et al., 2010), glutathione-casein was diluted in 50 mM carbonate buffer (pH9.6) and 200 ng/well were coated overnight at 4°C to 96 well plates (Nunc PolySorp). After blocking with 180 µl/well casein blocking buffer (CBB, 0.2% casein in PBST: 0.05% Tween-20 in PBS) for 1 hr at 37°C, the plate was incubated with the respective antigen (bacterial lysate containing the GST-antigen-SV40-tag fusion protein) for 1 hr at RT. *Mastomys* sera diluted 1:50 in CBB containing GST-SV40-tag were incubated for 1 hr at RT to remove unspecific reaction against bacterial proteins or the GST-SV40-tag fusion protein. Afterwards, ELISA plates were washed four times with PBST and incubated with pre-incubated sera for 1 hr at RT. After washing four times, 100 µl/well HPR-conjugated goat anti-mouse IgG (H+L) antibody (Promega, 1:10,000 in CBB) were applied for 1 hr at RT. Antibodies were quantified colorimetrically by incubating with 100 μl/well substrate buffer for 8 min (0.1 mg/ml tetramethylbenzidine and 0.006% H_2_O_2_ in 100 mM sodium acetate, pH6.0). The enzymatic reaction was stopped with 50 μl/well 1 M sulfuric acid. The absorption was measured at 450 nm in a microplate reader (Labsystems Multiskan, Thermo Fisher Scientific). To calculate the serum reactivity against the respective antigen, sera were tested in parallel against the GST-SV40-tag fusion protein and the reactivity was subtracted from the reactivity against the GST-antigen-SV40-tag. Each ELISA was performed in duplicates at least. The cut-offs were calculated individually for each antigen by measuring sera of virus-free animals.

### VLP-ELISA

VLP-ELISAs were performed as described elsewhere (Vinzón et al., 2014). Briefly, 100 ng/well purified high quality L1_SHORT_-VLPs were coated overnight in 50 mM carbonate buffer pH9.6 and blocked with CBB the next day. After incubation for 1 hr at RT with three-fold dilutions of sera in CBB, plates were washed four times with PBST and incubated with goat anti-mouse IgG-HRP (1:10,000 in CBB). After four washes, color development and measurement was performed as described for the GST-ELISA. Antibody titer represents the last reciprocal serum dilution above the blank.

### Denatured ELISAs

VLPs and GST fusion antigens were denatured at 95°C for 10 min in coating buffer (50 mM carbonate buffer pH9.6) prior to coating to ELISA plates overnight at 37°C. For the denatured VLP-ELISA, further steps were carried out according to the VLP-ELISA protocol described above. Denatured GST-ELISA antigens were then directly coated onto the ELISA plates overnight at 37°C and further steps (blocking, washing, incubation with sera, color reaction) were carried out according to the GST-ELISA protocol. The five monoclonal MnPV anti-L1_SHORT_ antibodies mAb2E2, mAb2D6, mAb2D11, mAb5E5 and mAb3H8 (reactivities shown in Supplementary file 2) were generated via hybridoma technique from BALB/c mice vaccinated with MnPV L1_SHORT_-VLPs and were used together with a *Mastomys* serum mix (sera from five tumor-bearing animals) as controls for denaturation conditions.

### Peptide arrays

The peptide array (PEPperPRINT GmbH, Germany) was produced as previously described (Stadler et al., 2008). The amino acid sequence of MnPV L1 was elongated with neutral GSGSGSG linkers at the C- and N-termini to avoid truncated peptides. Elongated antigen sequences were translated into 15 aa peptides with peptide-peptide overlaps of 14 aa. The resulting peptide microarray contained 530 different overlapping L1 peptides printed in duplicates and framed by additional HA (YPYDVPDYAG, 86 spots) control peptides. The peptide array was incubated for 10 min in PBST, followed by incubation in Rockland Blocking Buffer MB-070 (RBB; Rockland Immunochemicals, USA) for 1 hr. After short rinsing with PBST, the array was incubated for 16 hr at 4°C with *Mastomys* serum mix at a dilution of 1:300 in 10% RBB in PBST. The array was washed three times for 1 min with PBST and then incubated for 1 hr at RT with 0.2 µg/ml 10% RBB in PBST goat anti-mouse IgG (Fc) DyLight680 (Rockland Immunochemicals, USA). Subsequently, the array was washed three times for 1 min with PBST and rinsed with 1 mM TRIS-HCL pH7.4. As peptide controls, HA peptide spots were stained with monoclonal mouse-anti-HA IgG antibody (12CA5, kindly provided by Dr. G. Moldenhauer, DKFZ) conjugated with DyLight800 (Lightning-Link, Innova Biosciences, UK), followed by washing as described above. The antibody was diluted to 1 µg/ml in 10% RBB in PBST and staining was performed for 1 hr at RT in the dark followed by washing as described above. After drying of the array, fluorescence images were acquired with an Odyssey Infrared Imager (LICOR, USA) at a resolution of 21 µm. Scanner sensitivity was set to 7.0 for the 700 and 800 nm channels respectively, the focal plane was set to +0.8 mm. Quantification of spot intensities, based on 16-bit gray scale tiff files and microarray image analysis, via PepSlide Analyzer (SICASYS Software GmbH, Germany). A software algorithm breaks down fluorescence intensities of each spot into raw, foreground and background signal, and calculates averaged median foreground intensities and spot-to-spot deviations of spot duplicates. Averaged spot intensities of the assays with the sample were plotted against the antigen sequence from N- to C-terminus to visualize overall spot intensities and signal-to-noise ratios (intensity plot).

### Pseudovirion production

Pseudovirions were produced as previously described (Buck and Thompson, 2007). 293TT cells were co-transfected by calcium phosphate transfection with plasmids encoding humanized MnPV L1 isoforms (L1_LONG_, L1_MIDDLE_ and L1_SHORT_), L2 and a reporter plasmid encoding Gaussia luciferase. The 2^nd^ and 3^rd^ ATG of L1_LONG_ and the 2^nd^ ATG of L1_MIDDLE_ were mutated to GCG (alanine) to exclusively guarantee L1_LONG_ or L1_MIDDLE_ expression. Transfected cells were incubated for 48 hr, harvested and resuspended in an equal volume of PBS and supplemented with 0.5% Brij 58 (Sigma) and 1% RNase A/T1 mix (Thermo Fisher Scientific). Cells were lysed for 24 hr under rotation at 37°C to allow pseudovirion maturation prior to adjustment with 5 M NaCl to 0.85 M NaCl and treatment with 700 U Benzonase (Merck) for 1 hr at 37°C. For purification, the lysate was transferred on top of a three-step gradient of 27%, 33% and 39% Iodixanol (Optiprep, Sigma) diluted in 0.8 M NaCl/DPBS. and centrifuged at 37,000 rpm for 5 hr at 16°C in a swinging bucket rotor. Fractions of 500 µl each were collected in siliconized LoBind tubes (Eppendorf) and quantity and quality of pseudovirions in each fraction was assessed by electron microscopy (EM) and Gaussia luciferase reporter activity after infection of HeLaT cells.

### Pseudovirion-based neutralization assay

As previously described (Buck et al., 2005a), animal sera (in duplicates, initial dilution 1:60 in medium) were subjected to 1:3 serial dilutions in 96-well cell culture plates (Greiner Bio-One GmbH). Then, the sera were mixed with 40 µl of diluted pseudovirions and incubated for 15 min at RT. Then, 50 µl of 2.5 × 10^5^ HeLaT cells/ml were seeded to the pseudovirion-serum mixture and cultured for 48 hr at 37°C. The activity of secreted Gaussia luciferase was measured 15 min after adding coelenterazine substrate and Gaussia glow juice (PJK Biotech, Germany) according to the manufacturer’s instructions in a microplate luminometer reader (Synergy 2, BioTek). The neutralization titer represents the reciprocal of the highest dilution that reduces the signal by at least 50%.

### Construction of pFBDM plasmids and Multibac plasmids

Two copies of MnPV wildtype L1_LONG_, L1_MIDDLE_ and L1_SHORT_ were inserted into the Multibac vector pFBDM using *EcoRI/HindIII* and *XmaI/SphI*. Comparable to the VLP production, to ensure that only L1_LONG_ and L1_MIDDLE_ are expressed in the Multibac expression system, the 2^nd^ and 3^rd^ ATG start codons of L1_LONG_ and the 2^nd^ ATG of L1_MIDDLE_ were mutated to GCG (alanine). The recombinant MultiBac bacmids were generated by electroporation (1.8 kV pulse) of DH10MultiBac^Cre^* E. coli* with the generated plasmids, followed by selection with antibiotics and blue/white screening (Fitzgerald et al., 2006). Recombinant MultiBac bacmids were isolated by QIAGEN Plasmid Mini Kit followed by ethanol precipitation.

### VLP production and purification

Recombinant baculoviruses were generated as previously described (Vinzón et al., 2014) with some modifications. One µg of MultiBac bacmid containing L1_LONG_, L1_MIDDLE_ or L1_SHORT_ was diluted in 1 ml transfection buffer (25 mM Hepes, 125 mM CaCl_2_, 140 mM NaCl, pH7.2) and added dropwise to Sf9 cells. After incubation at 27°C for 5 hr, cells were washed twice and then cultured for 6 days in supplemented TNM-FH medium (Sigma). One ml supernatant was used for generation of a high-titer baculovirus stock by infecting 2 × 10^6^ Sf9 cells in a T25 flask followed by virus amplification for 6 days. This step was repeated with the obtained supernatant two times with increasing cell numbers (3 ml supernatant for 1 × 10^7^ cells in a T75 flask and 5 ml supernatant for 2.5 × 10^7^ cells in a T175 flask). TN-High Five cells were cultivated to a density of 2.5 × 10^6^/ml in 250 ml suspension culture, which were then pelleted and resuspended in 42 ml EX-CELL 405 serum-free medium (Sigma) and 8 ml high titer virus stock. The cells were shaken at a low speed for 1 hr at RT and then incubated within 250 ml final volume of medium for 3 days at 27°C. Cell pellets were harvested by centrifugation (3000 rpm for 10 min at 4°C in a Sorvall GS-3 rotor) and washed in pre-chilled PBS for two times. Dry pellets were resuspended in 10 ml VLP extraction buffer (5 mM MgCl_2_, 5 mM CaCl_2_, 150 mM NaCl, 0.01% Triton X-100 and 20 mM Hepes pH7.4) containing 200 μl 100 mM PMSF, and then followed by three times sonication. A two-step gradient consisting of 7 ml of 40% sucrose on top of 7 ml CsCl solution was prepared. Clear cell lysate was obtained by centrifugation (10,000 rpm for 10 min at 4°C in a Sorvall F-28/50 rotor) and carefully loaded onto the top of the CsCl layer. After centrifugation (27,000 rpm for 3 hr at 10°C in SW-31Ti rotor), the interphase between sucrose and CsCl together with the complete CsCl layer was transferred into a Quickseal tube. The fractions were collected in 1 ml aliquot after 16 hr centrifugation at 48,000 rpm at 20°C in a Beckman 70Ti rotor and analyzed by Coomassie blue dye and Western blot. Small aliquots from the fraction with highest and lowest protein yield were dialyzed against H_2_O on a membrane filter and analyzed by EM.

### Electron microscopy (EM)

VLP and PsV preparations or tissue were fixed with buffered aldehyde solution (2% formaldehyde, 2% glutaraldehyde, 1 mM MgCl_2_, 2% sucrose in 100 mM calcium cacodylate, pH7.2), followed by post-fixation in buffered 1% OsO_4_, graded dehydration with ethanol and resin-embedding in epoxide (12 g glycid ether, 6.5 g NMA, 6.5 g DDSA, 400 μl DMP30, all from Serva, Germany). Ultrathin sections at nominal thickness 60 nm and contrast-stained with lead-citrate and Uranylacetate were observed in a Zeiss EM 910 at 100 kV (Carl Zeiss, Oberkochen, Germany) and micrographs were taken with image-plates, scanned at 30 µm resolution (Ditabis micron, Pforzheim, Germany).

### Transfection of mammalian cells, SDS-PAGE and western blotting

Variants of L1 ORFs were cloned into pPK-CMV-E3 expression plasmids. For exclusive and strong eukaryotic expression of the respective L1 isoform, viral codons were humanized, unwanted start codons mutated (L1_SHORT_ starts from 3^rd^ ATG; L1_MIDDLE_ starts from 2^nd^ ATG, 3^rd^ ATG mutated; L1_LONG_ starts from 1^st^ ATG, 2^nd^ and 3^rd^ ATG mutated) and the ORFs were cloned downstream of an artificial Kozak sequence. Alternatively, the complete genuine viral L1 ORF encoding start codons and Kozak sequences of all isoforms was cloned and termed pPK-CMV_MnPV-vL1.

MaFi132 cells (400,000 cells/10 cm dish) were transfected with 5 µg pPK-CMV_MnPV-L1_SHORT_ or either 10 µg pPK-CMV_MnPV-L1_MIDDLE_, pPK-CMV_MnPV-L1_LONG_ or pPK-CMV_vL1 24 hr after seeding using TurboFect (Thermo Fisher Scientific) according to the manufacturer’s protocol. Cells were collected 48 hr after transfection, washed in PBS and lysed for 30 min on ice in 1.25x Laemmli buffer (78 mM Tris pH6.8, 2.5% SDS, 6.25% glycerol, 0.125% bromophenol blue, 2.5% β-mercaptoethanol). Lysates were then heated at 95°C for 5 min, chilled on ice and treated with 100 U/ml Benzonase (Millipore) for 5 min at RT. Protein concentrations were measured using a NanoDrop spectrophotometer. Forty μg lysate/lane were loaded to 8% SDS-PAGE. To guarantee complete denaturation of the samples, additional DTT and β-mercaptoethanol were added to a final concentration of 100 mM and 4%, respectively, prior to incubation for 1 hr at RT and heating at 95°C for 10 min. After blotting, proteins were detected with anti-HA (3F10, 1:1000, Roche), anti-vinculin (7F9, 1:4000, Santa Cruz), anti-MnPV-L1_SHORT_ (mAb 2D11, 1:5) or *Mastomys* serum mix (1:1000) prior to detection with goat anti-mouse-HRP (W4021, 1:10,000, Promega) or goat anti-rat-HRP (1:10,000, Jackson ImmunoResearch). For Coomassie staining, gels were incubated overnight in Coomassie stain and then destained in 20% methanol.

### Immunofluorescence stainings

MaFi132 cells were transfected with L1 isoforms as described above and seeded on glass cover slides after 24 hr. Additional 48 hr later the cells were washed with PBS and fixed for 10 min in 4% PFA. Cells were blocked in 10% goat serum/0.3% Triton X-100 in PBS for 1 hr and stained with anti-MnPV-L1_SHORT_ (2D11, 1:5), *Mastomys* serum mix (1:1000) or anti-HA (3F10, 1:1000, Roche) and the respective secondary goat anti-mouse or donkey anti-rat IgG (conjugated to AlexaFluor488, 1:1000, Invitrogen). Nuclei were stained with DAPI. Cover slides were mounted with Faramount Aqueous Mounting Medium (Dako) and imaged with a Cell Observer (Carl Zeiss).

### Immunohistochemistry (IHC)

Staining of formalin-fixed, paraffin-embedded tumors was performed as previously described (Hasche et al., 2017). Briefly, deparaffinized sections were heated in citrate buffer pH6.0 prior to blocking with 5% goat serum/5% FCS/1% BSA in PBS and incubation with primary antibodies (serum of a VLP-vaccinated *Mastomys* (Vinzón et al., 2014), serum of a mouse immunized with the N-terminal 31 aa of MnPV-L1_LONG_ in the OVX313 platform (Spagnoli et al., 2017) or the respective pre-immune sera, cross-reactive anti-L2 (K18L2) (Rubio et al., 2011) or serum of a guinea pig immunized with Gardasil9 (unpublished) overnight at 4°C. Detection of L1 isoforms was achieved with the Dako REAL Detection System, Peroxidase/AEC, Rabbit/Mouse. The color reaction with AEC/H_2_O_2_ substrate solution (Sigma) was stopped with distilled water followed by counterstaining with hemalum solution (Carl Roth, Karlsruhe, Germany). Fluorescence stainings were detected with anti-mouse-IgG1-Alexa594 or anti-guinea-pig-Alexa488 (Invitrogen) and nuclei were stained with DAPI. Sections were mounted with Dako Faramount Aqueous Mounting Medium.

### Statistical analysis

Data analyses and graphic representations were performed with GraphPad Prism 6.0 Software and the respective statistical test indicated in the figure legends at 95% confidence interval and an alpha level of 5% to assess significance. For time course analyses, individual time points were compared to the eight-week starting time point. The rate of L1_LONG_- and L1_SHORT_- positive animals was calculated and compared with a two-tailed McNemar’s test at an alpha level of 5% to assess significance.

## Data Availability

All data generated or analysed during this study are included in the manuscript and supporting files.

## Additional files

Supplementary file 1. Alignment of L1 amino acid sequences of MnPV and HPV6, 16 and 18.

Supplementary file 2. Binding properties of monoclonal antibodies against L1 isoforms.

Supplementary file 3. Prediction of ATG usage in MnPV late transcript Q.

Transparent reporting form
