# Supplementary material for: Expression of different L1 isoforms of Mastomys natalensis papillomavirus as mechanism to circumvent adaptive immunity
Source: eLife. 2020 Aug 4;9:e57626. doi: 10.7554/eLife.57626 (PMC7402679; doi:10.7554/eLife.57626)
Supplement: Supplementary file 1. [file elife-57626-supp1.docx]

**Sequence alignment of L1_SHORT_ proteins of MnPV and HPV6, 16 & 18**

CLUSTAL O(1.2.4) multiple sequence alignments

**BC loop**

MnPV-L1 MAYWLPNNQKLYLP-PAPVQRILSTDEFTTRTDIYYYASSDRLLTVGNPYYPILDG--DT 57

HPV18-L1 MALWRPSDNTVYLPPP-SVARVVNTDDYVTRTSIFYHAGSSRLLTVGNPYFRVPAGGGNK 59

HPV16-L1 MSLWLPSEATVYLP-PVPVSKVVSTDEYVARTNIYYHAGTSRLLAVGHPYFPIKKPNNNK 59

HPV6-L1 M--WRPSDSTVYVPPPNPVSKVVATDAYVTRTNIFYHASSSRLLAVGHPYFSIKRA--NK 56

* *.: .:*:* * * ::: ** :.:**.*:*:*.:.***:**:**: : :.

**BC loop** **DE loop**

MnPV-L1 VTVPKVSPNQYRVFRCKLPDPNRFAFGEKSVYDPEKQRLAWCIRGVEIARGQPLGIGITG 117

HPV18-L1 QDIPKVSAYQYRVFRVQLPDPNKFGLPDTSIYNPETQRLVWACAGVEIGRGQPLGVGLSG 119

HPV16-L1 ILVPKVSGLQYRVFRIHLPDPNKFGFPDTSFYNPDTQRLVWACVGVEVGRGQPLGVGISG 119

HPV6-L1 TVVPKVSGYQYRVFKVVLPDPNKFALPDSSLFDPTTQRLVWACTGLEVGRGQPLGVGVSG 116

:**** *****: *****:*.: :.*.::* .***.*. *:*:.******:*::*

**DE loop EF loop**

MnPV-L1 HPLYNRLEDVENPGKYPSAPGTDNRQNVGLDPKQTQMFIVGCVPAQGEHWSRALTCSNQV 177

HPV18-L1 HPFYNKLDDTESSHAATSNVSEDVRDNVSVDYKQTQLCILGCAPAIGEHWAKGTACKSRP 179

HPV16-L1 HPLLNKLDDTENASAYAANAGVDNRECISMDYKQTQLCLIGCKPPIGEHWGKGSPCTNVA 179

HPV6-L1 HPFLNKYDDVENSG-SGGNPGQDNRVNVGMDYKQTQLCMVGCAPPLGEHWGKGKQCTNTP 175

**: *: :*.*. . . * * :.:* ****: ::** * ****.:. *..

**EF loop**

MnPV-L1 VKKGDCPPIQRMSGMIEDGDMGDIGYGNLDFRVLQENKSEVPLEVVDSICKYPDYLGMSK 237

HPV18-L1 LSQGDCPPLELKNTVLEDGDMVDTGYGAMDFSTLQDTKCEVPLDICQSICKYPDYLQMSA 239

HPV16-L1 VNPGDCPPLELINTVIQDGDMVDTGFGAMDFTTLQANKSEVPLDICTSICKYPDYIKMVS 239

HPV6-L1 VQAGDCPPLELITSVIQDGDMVDTGFGAMNFADLQTNKSDVPIDICGTTCKYPDYLQMAA 235

:. *****:: . :::**** * *:* ::* ** .*.:**::: : ******: *

**FG loop**

MnPV-L1 ETHGNSCFFYARQARLYSRHFFNRAGVQGETVPESLYKKGKDGQAQSTLALATYSGTPSG 297

HPV18-L1 DPYGDSMFFCLRREQLFARHFWNRAGTMGDTVPQSLYIKGTGMRA--SPGSCVYSPSPSG 297

HPV16-L1 EPYGDSLFFYLRREQMFVRHLFNRAGTVGENVPDDLYIKGSGSTA--NLASSNYFPTPSG 297

HPV6-L1 DPYGDRLFFFLRKEQMFARHFFNRAGEVGEPVPDTLIIKGSGNRT--SVGSSIYVNTPSG 293

: :*: ** *: ::: **::**** *: **: * **.. : . . . * :***

**HI loop**

MnPV-L1 SLVSSDAVLFNRPYWLERAQGQNNGILWNNDLFVTVLDNTRGTHFSISIATQ--DENDYT 355

HPV18-L1 SIVTSDSQLFNKPYWLHKAQGHNNGVCWHNQLFVTVVDTTRSTNLTICASTQSPVPGQYD 357

HPV16-L1 SMVTSDAQIFNKPYWLQRAQGHNNGICWGNQLFVTVVDTTRSTNMSLCAAIST-SETTYK 356

HPV6-L1 SLVSSEAQLFNKPYWLQKAQGHNNGICWGNQLFVTVVDTTRSTNMTLCASVTT-S-STYT 351

*:*:*:: :**:****.:***:***: * *:*****:*.**.*::::. : *

**HI loop**

MnPV-L1 ASNYKQYTRHVEEFELEFIFQLVKINLSTEVLAYLHGMDPSILDNWNLTLGPPNDGSLAD 415

HPV18-L1 ATKFKQYSRHVEEYDLQFIFQLCTITLTADVMSYIHSMNSSILEDWNFGVPPPPTTSLVD 417

HPV16-L1 NTNFKEYLRHGEEYDLQFIFQLCKITLTADVMTYIHSMNSTILEDWNFGLQPPPGGTLED 416

HPV6-L1 NSDYKEYMRHVEEYDLQFIFQLCSITLSAEVMAYIHTMNPSVLEDWNFGLSPPPNGTLED 411

:.:*:* ** **::*:***** .*.*:::*::*:* *: ::*::**: : ** :* *

MnPV-L1 KYRFIESLATKCPDN-VEVTKPDPYKGRIFWNIDLTERLTADLDQFSLGRKFLYQHARIS 474

HPV18-L1 TYRFVQSVAITCQKDAAPAENKDPYDKLKFWNVDLKEKFSLDLDQYPLGRKFLVQAGLRR 477

HPV16-L1 TYRFVTSQAIACQKHTPPAPKEDPLKKYTFWEVNLKEKFSADLDQFPLGRKFLLQAGLKA 476

HPV6-L1 TYRYVQSQAITCQKPTPEKEKPDPYKNLSFWEVNLKEKFSSELDQYPLGRKFLLQSGYRG 471

.**:: * * * . : ** . **:::*.*::: :***: ****** * .

MnPV-L1 NRKRSLPASRNGGGTSSSSTKRRK-K---- 499

HPV18-L1 KPTIGPRKRSAPSATTSSKPAKRVRVRARK 507

HPV16-L1 KPKFTLGKRKATPTTSSTSTTAKRKKRKL- 505

HPV6-L1 RSSIRTGVKRPAVSKASAAPKRKRAKTKR- 500

. . .:*: :

Marked areas based on: Zhang, X., Li, S., Modis, Y., Li, Z., Zhang, J., Xia, N., and Zhao, Q. (2016). Functional assessment and structural basis of antibody binding to human papillomavirus capsid. Reviews in medical virology *26*, 115-128.
