# Supplementary material for: Expression of different L1 isoforms of Mastomys natalensis papillomavirus as mechanism to circumvent adaptive immunity
Source: eLife. 2020 Aug 4;9:e57626. doi: 10.7554/eLife.57626 (PMC7402679; doi:10.7554/eLife.57626)
Supplement: Supplementary file 2. [file elife-57626-supp2.docx]

**Supplementary File 3 Binding properties of monoclonal antibodies against L1 isoforms**

|  | Native conditions | | Denatured conditions | | PBNA |
| --- | --- | --- | --- | --- | --- |
| Antibody | **GST-L1_LONG_** | **GST-L1_SHORT_** | **GST-L1_LONG_** | **GST-L1_SHORT_** |  |
| mAb 2E2 | **-** | ******** | **-** | **-** | ******* |
| mAb 2D6 | ******* | ******* | ******* | ******* | **-** |
| mAb 2D11 | ***** | ******** | **-** | **-** | ******* |
| mAb 5E5 | ****** | ****** | ******** | ******* | **-** |
| mAb 3H8 | **-** | ******** | **-** | **-** | ******* |

**ELISAs: ******: OD_450_ > 2 ; *******: 1 <OD_450_ < 2 ; ******: 0.1 < OD_450_ < 1 ; *****: 0.05 < OD_450_ < 0.1 ; **-**: OD_450_ = Blank

**PBNA: *****: 100% neutralization of PsVs with undiluted hybridoma supernatant
