# Supplementary material for: Expression of different L1 isoforms of Mastomys natalensis papillomavirus as mechanism to circumvent adaptive immunity
Source: eLife. 2020 Aug 4;9:e57626. doi: 10.7554/eLife.57626 (PMC7402679; doi:10.7554/eLife.57626)
Supplement: Supplementary file 3. [file elife-57626-supp3.docx]

**Identification of initiation codons in Transcript Q [710...808,3145…7322]**

**calculated with: https://atgpr.dbcls.jp**

ACAAGCACACCAGATCCGTGGGCTAGAACATCTACTGCTTGACGGGCTAAGAGTGATCTGTCCGCGGTGTAACCAGAAGAATGGAAGATCTTGAAGAAGCTCTACACCGCAGACCGGATTTCCTAGAGGGGATCCGGTACGCCTTCACGGGAATACCACCACAGGACTGCCCATACCGCTTCGGAACAGCAGCAGCAACCAGATATTACTACGAGAGGGAAGAGGAGACTATCCAGACGGCGCAGCCCCGCGAGACGAGGAGGTACTACCAGGGGCCAACACCGACGCCCAGGTCTCTATCTCCCCCCATCTACCGTCCCCCGCCAAGCTACGAAGAGTCGAGGAGGAGGAGGAAGCTAAGGCGCCGCCAAGACGGGCGAGTCAAATACCCCGCGTCTCCCTACAGGACAAAACCACCGGGGGAAACCAGCAGCGACGACGAAGACGAGGGGAGAGGGGGGCACGAACCCCGTCCCCAGAGACGACTGCCCAGAGGCCTAAGAGACCGCGGAGAGCGTGCACCCGAAAGGAGGAGACCCCCAGTTCAGGAGGGGGAGGAGGACGTGGACGGCGTAGGGGCCTTGCTGGACGACCTGAAGCTGTACCAAGAACCACCTGGAGACCCAGTGGAGGACTCGGACTCCCCAGGCAGTCGTCTACCCCCGCCCCGCCAGACCTATCTCGGTACGACTCTACCCGGTTACAGGTGGACGCGGAGAGCAGCCCTCCTAGGACACCCAGACCGGCCCCCACTCTCGTGGCAGAGTGCACTCCTGGGAGACCTTCTCCGCAGACTGGAAGCGGACAGCAAGCACTGGGAGAACCGCCTTCTCGGCCTTCACGCGGACATTGCCGCGACCCTCGGACTGCCTGCCTTTTGATCATCAAAGGATCATCAAATCAGGTTAAGTGCTTGCGATTTAGACTTAAATCCTGGCATCACAGCCTGTTTTCCTACATCAGCACCACATGGCAGTGGGTTCCCTCAGTAGGAAGTAATAGGATTGGACGGTCACGCATTCTGGTGATGTGTGAGGACTCAGCGCAGATGGACAGATTCCTATGTACTGTTAAGATCCCTGCTGTTATGACAGTTGAACAGTGCAGCATGGCGTCTGTCTGATGCCCCCCCCCCTCGCATAACATACTAACGCACACTGCAATAAAGTTTTTCCTTTACACAGTACTAACCTACTAATATTAGCATGTCTAGAAGGAGAAAGCGACATACACGAGTCCCTCGTGACTCGGCCACTCACATATATCAAACATGTAAGCAGGCAGGCACATGTCCGCCTGATGTAGTTAATAAAGTTGAAGGCACAACCACAGCTGATAAGATTCTTCAATATGGCGGGGCGGCTGTATTCCTCGGTGGCCTTGGTATTGGTACAGGTAGGGGAAGTGGTGGTGCAACAGGGTATGTACCGGTCGGCGAGACACCTGGTATTTCCGTGGGTGCAAGACCAGTTCCTCGACCTAATGTGCCCTTAGAAACTGTTGGTCCCCAGGACCTGTTTCCTGTGGATGCCATTAGGCCTACTGATCCTTCGGTGATTGATGTCGCCAGTGTGCCTACTCCCACTGACACCTCTATTAATGTACCCGAGGTGGAGGTCATTGCTGAGATCCACCCTGTACCTCCTGACGGTCCCTCCAACACACCAACAACCACAATTAACACATCAGGCTCAGGGGATGCAGCCATATTAGAGGTAGCTCCTGAACCATCCCCAGCCGTCAGGACTCGGTGGAGAGCTAGCAAGACAACCTTCCATAATCCTGCCTTTCACAGCTTCTCCTCTACTGGTTCAACTGTAGGCGAGGCCACAGGTATGGACAATATTGTTGTTTACAGCGGTAGTGGGGGGAGGACGATAGGTGGGGACAGCATAGAGCTTATGCCCTTTACTAGCAGTGATACCCTAGATTTAAGTATTGTGGAGGAGACCTCCTTTGGAGGTAGGACCAGCACACCACGAACCAAGCCCCTCCCTTCTCGGTTGCCTTCCCGGAGGTATTATGAATATAGAGAAAGCAGTCTTGGTGAGTTATGGTCACCTAGGAGGGCTATGGGTCCCACGTATATAAATCCTGCCTTTGAAGCTGAGGATAGTATCCTTTTTCCTGAAAGTAGCATGCAGGCCGCTAATCCAGATTACACAGGCATTACCAGGCTTGGTCATCTCTTTGGTACTGAGCAGGGTGGCCGTGTCCGTATTGGTCGTCTGGGACAAAAGACATCCCTGCACACACGCAGCGGTATGGCAATAGGCCCTAAGGCATACTTTTATAAGGACATTTCTAGCATTTCTGTTGTCCCAGAGGAGAGTATAGAACTCAGCACCTATACCTCAGCTGCCCCTTTGGGTGAGGATGCAGGTATAATAGTGGAGGACTCTATGGAGGGTTCTTTTGACAATATCACCCTCAGTTCTTGGAGTCATGGATCCATGGACGGGCTTCTTGAGGATGATGCTAGTTATGATTTTCACGGCCACCTGGTGTGGGGAACACGCCGTAGCTCTAAGCAAATAAGCATGCCATTCCGCCGGTCGTGGTATCCTGAAACTGCTGTGTACGTGCAGGAGGGTGGGTCTGTAATGGATCCTGAGGCTTCTGCAGAGCTGGTTCCCAGTAGGGACAGTGCTCGTCCCCATGTCATATATAGGGGCTATAATGGGACGGACTATTATCTACACCCGTCATTGTCCAGACGCAGGCGTAAGCGCAGGCATATCTATTTTTCAGATGGCGTACTGGCTGCCTAATAACCAGAAGTTGTACCTGCCCCCGGCCCCGGTGCAGCGCATACTGTCTACAGATGAATTTACTACACGAACAGACATATATTACTATGCTAGTAGTGACAGGTTATTAACTGTTGGTAATCCATATTATCCTATACAGGATGGGGATACTGTTACTGTTCCTAAGGTCAGTCCTAATCAATACAGGGTGTTCCGTTGTAAATTACCGGACCCTAACCGGTTTGCATTTGGTGAGAAGTCGGTTTACGACCCTGAGAAGCAACGGCTTGCATGGTGTATACGGGGAGTGGAGATAGCTCGTGGCCAACCTCTGGGAATAGGGATTACTGGGCATCCCCTATATAACAGGCTAGAGGATGTGGAGAACCCTGGAAAGTATCCATCTGCTCCGGGCACGGACAATAGACAAAATGTAGGCCTTGATCCGAAGCAGACTCAGATGTTTATTGTCGGTTGTGTACCTGCACAGGGTGAGCACTGGAGTAGAGCACTTACCTGCAGCAATCAGGTGGTTAAGAAGGGTGACTGTCCACCTATTCAGCGGATGTCTGGGATGATTGAGGATGGTGACATGGGGGACATAGGTTATGGCAACTTAGACTTCCGAGTGTTGCAGGAAAACAAGTCAGAGGTTCCCCTCGAGGTAGTTGACTCTATCTGTAAGTACCCCGATTATTTAGGAATGTCCAAGGAAACCCACGGCAACTCATGCTTCTTCTATGCTAGGCGGGAGCAATTATACAGCAGGCACTTCTTTAACCGTGCAGGTGTTCAGGGTGAGACTGTGCCGGAGTCATTATACAAGAAGGGCAAGGATGGACAGGCACAGAGCACACTGGCACTAGCTACATACTCAGGGACTCCGTCAGGGTCACTAGTGTCATCTGATGCTGTACTGTTCAACCGTCCATACTGGCTTGAGAGGGCACAAGGACAAAACAATGGCATTCTGTGGAATAATGATTTGTTCGTGACCGTGCTGGACAACACTCGTGGGACCCATTTCTCCATCAGCATTGCTACACAGGATGAAAATGATTACACCGCCTCAAACTACAAGCAATATACTCGACATGTTGAAGAATTTGAGCTTGAATTTATTTTCCAACTGGTTAAGATCAACCTTTCTACTGAGGTGCTAGCATACCTGCATGGGATGGACCCATCTATACTGGATAACTGGAACTTGACTCTGGGACCCCCCAATGATGGTAGCCTTGCTGATAAGTACAGATTTATAGAATCCCTTGCTACAAAATGCCCTGACAATGTGGAAGTCACTAAGCCTGATCCCTACAAAGGACGGATATTCTGGAACATTGACCTGACTGAAAGACTGACAGCTGATCTGGACCAATTCTCACTTGGACGGAAGTTCCTCTACCAGCACGCGCGAATTTCAAACCGTAAACGGTCCCTTCCTGCTTCCAGAAACGGCGGCGGAACCTCCTCCTCTTCCACCAAGCGGAGAAAAAAATAGTTGGAATAAAGACTGCTGACACTGCA

**Color code: E1^E4 L2 L1_LONG_ L1_MIDDLE_ L1_SHORT_**

| **No of ATG from 5'end** | **Reli**  **abil**  **ity (RI)** | **Frame** | **Identity to Kozak rule A/GXXATGG** | **Start (bp)** | **Finish (bp)** | **ORF Length (aa)** | **Stop codon found?** | **Protein Sequence** |
| --- | --- | --- | --- | --- | --- | --- | --- | --- |
| **1** | **0.43** | 3 | **AXXATGG** | 81 | 878 | 266 | Yes | MEDLEEALHRRPDFLEGIRYAFTGIPPQDCPYRFGTAAATRYYYEREEETIQTAQPRETRRYYQGPTPTPRSLSPPIYRPPPSYEESRRRRKLRRRQDGRVKYPASPYRTKPPGETSSDDEDEGRGGHEPRPQRRLPRGLRDRGERAPERRRPPVQEGEEDVDGVGALLDDLKLYQEPPGDPVEDSDSPGSRLPPPRQTYLGTTLPGYRWTRRAALLGHPDRPPLSWQSALLGDLLRRLEADSKHWENRLLGLHADIAATLGLPAF |
| **9** | **0.42** | 3 | **AXXATGt** | 1206 | 2768 | 521 | Yes | MSRRRKRHTRVPRDSATHIYQTCKQAGTCPPDVVNKVEGTTTADKILQYGGAAVFLGGLGIGTGRGSGGATGYVPVGETPGISVGARPVPRPNVPLETVGPQDLFPVDAIRPTDPSVIDVASVPTPTDTSINVPEVEVIAEIHPVPPDGPSNTPTTTINTSGSGDAAILEVAPEPSPAVRTRWRASKTTFHNPAFHSFSSTGSTVGEATGMDNIVVYSGSGGRTIGGDSIELMPFTSSDTLDLSIVEETSFGGRTSTPRTKPLPSRLPSRRYYEYRESSLGELWSPRRAMGPTYINPAFEAEDSILFPESSMQAANPDYTGITRLGHLFGTEQGGRVRIGRLGQKTSLHTRSGMAIGPKAYFYKDISSISVVPEESIELSTYTSAAPLGEDAGIIVEDSMEGSFDNITLSSWSHGSMDGLLEDDASYDFHGHLVWGTRRSSKQISMPFRRSWYPETAVYVQEGGSVMDPEASAELVPSRDSARPHVIYRGYNGTDYYLHPSLSRRRRKRRHIYFSDGVLAA |
| **36** | **0.38** | 1 | **cXXATGt** | 2659 | 4248 | 530 | Yes | MSYIGAIMGRTIIYTRHCPDAGVSAGISIFQMAYWLPNNQKLYLPPAPVQRILSTDEFTTRTDIYYYASSDRLLTVGNPYYPIQDGDTVTVPKVSPNQYRVFRCKLPDPNRFAFGEKSVYDPEKQRLAWCIRGVEIARGQPLGIGITGHPLYNRLEDVENPGKYPSAPGTDNRQNVGLDPKQTQMFIVGCVPAQGEHWSRALTCSNQVVKKGDCPPIQRMSGMIEDGDMGDIGYGNLDFRVLQENKSEVPLEVVDSICKYPDYLGMSKETHGNSCFFYARREQLYSRHFFNRAGVQGETVPESLYKKGKDGQAQSTLALATYSGTPSGSLVSSDAVLFNRPYWLERAQGQNNGILWNNDLFVTVLDNTRGTHFSISIATQDENDYTASNYKQYTRHVEEFELEFIFQLVKINLSTEVLAYLHGMDPSILDNWNLTLGPPNDGSLADKYRFIESLATKCPDNVEVTKPDPYKGRIFWNIDLTERLTADLDQFSLGRKFLYQHARISNRKRSLPASRNGGGTSSSSTKRRKK |
| 25 | 0.26 | 3 | AXXATGc | 2139 | 2768 | 210 | Yes | MQAANPDYTGITRLGHLFGTEQGGRVRIGRLGQKTSLHTRSGMAIGPKAYFYKDISSISVVPEESIELSTYTSAAPLGEDAGIIVEDSMEGSFDNITLSSWSHGSMDGLLEDDASYDFHGHLVWGTRRSSKQISMPFRRSWYPETAVYVQEGGSVMDPEASAELVPSRDSARPHVIYRGYNGTDYYLHPSLSRRRRKRRHIYFSDGVLAA |
| 13 | 0.17 | 1 | AXXATGG | 1351 | 1491 | 47 | Yes | MAGRLYSSVALVLVQVGEVVVQQGMYRSARHLVFPWVQDQFLDLMCP |
| 20 | 0.17 | 3 | GXXATGG | 1836 | 2768 | 311 | Yes | MDNIVVYSGSGGRTIGGDSIELMPFTSSDTLDLSIVEETSFGGRTSTPRTKPLPSRLPSRRYYEYRESSLGELWSPRRAMGPTYINPAFEAEDSILFPESSMQAANPDYTGITRLGHLFGTEQGGRVRIGRLGQKTSLHTRSGMAIGPKAYFYKDISSISVVPEESIELSTYTSAAPLGEDAGIIVEDSMEGSFDNITLSSWSHGSMDGLLEDDASYDFHGHLVWGTRRSSKQISMPFRRSWYPETAVYVQEGGSVMDPEASAELVPSRDSARPHVIYRGYNGTDYYLHPSLSRRRRKRRHIYFSDGVLAA |
| **37** | **0.17** | 1 | **AXXATGG** | 2680 | 4248 | 523 | Yes | MGRTIIYTRHCPDAGVSAGISIFQMAYWLPNNQKLYLPPAPVQRILSTDEFTTRTDIYYYASSDRLLTVGNPYYPIQDGDTVTVPKVSPNQYRVFRCKLPDPNRFAFGEKSVYDPEKQRLAWCIRGVEIARGQPLGIGITGHPLYNRLEDVENPGKYPSAPGTDNRQNVGLDPKQTQMFIVGCVPAQGEHWSRALTCSNQVVKKGDCPPIQRMSGMIEDGDMGDIGYGNLDFRVLQENKSEVPLEVVDSICKYPDYLGMSKETHGNSCFFYARREQLYSRHFFNRAGVQGETVPESLYKKGKDGQAQSTLALATYSGTPSGSLVSSDAVLFNRPYWLERAQGQNNGILWNNDLFVTVLDNTRGTHFSISIATQDENDYTASNYKQYTRHVEEFELEFIFQLVKINLSTEVLAYLHGMDPSILDNWNLTLGPPNDGSLADKYRFIESLATKCPDNVEVTKPDPYKGRIFWNIDLTERLTADLDQFSLGRKFLYQHARISNRKRSLPASRNGGGTSSSSTKRRKK |
| **38** | **0.17** | 1 | **cXXATGG** | 2752 | 4248 | 499 | Yes | MAYWLPNNQKLYLPPAPVQRILSTDEFTTRTDIYYYASSDRLLTVGNPYYPIQDGDTVTVPKVSPNQYRVFRCKLPDPNRFAFGEKSVYDPEKQRLAWCIRGVEIARGQPLGIGITGHPLYNRLEDVENPGKYPSAPGTDNRQNVGLDPKQTQMFIVGCVPAQGEHWSRALTCSNQVVKKGDCPPIQRMSGMIEDGDMGDIGYGNLDFRVLQENKSEVPLEVVDSICKYPDYLGMSKETHGNSCFFYARREQLYSRHFFNRAGVQGETVPESLYKKGKDGQAQSTLALATYSGTPSGSLVSSDAVLFNRPYWLERAQGQNNGILWNNDLFVTVLDNTRGTHFSISIATQDENDYTASNYKQYTRHVEEFELEFIFQLVKINLSTEVLAYLHGMDPSILDNWNLTLGPPNDGSLADKYRFIESLATKCPDNVEVTKPDPYKGRIFWNIDLTERLTADLDQFSLGRKFLYQHARISNRKRSLPASRNGGGTSSSSTKRRKK |
| 51 | 0.15 | 1 | GXXATGt | 3454 | 4248 | 265 | Yes | MSKETHGNSCFFYARREQLYSRHFFNRAGVQGETVPESLYKKGKDGQAQSTLALATYSGTPSGSLVSSDAVLFNRPYWLERAQGQNNGILWNNDLFVTVLDNTRGTHFSISIATQDENDYTASNYKQYTRHVEEFELEFIFQLVKINLSTEVLAYLHGMDPSILDNWNLTLGPPNDGSLADKYRFIESLATKCPDNVEVTKPDPYKGRIFWNIDLTERLTADLDQFSLGRKFLYQHARISNRKRSLPASRNGGGTSSSSTKRRKK |
| 49 | 0.14 | 1 | GXXATGG | 3343 | 4248 | 302 | Yes | MGDIGYGNLDFRVLQENKSEVPLEVVDSICKYPDYLGMSKETHGNSCFFYARREQLYSRHFFNRAGVQGETVPESLYKKGKDGQAQSTLALATYSGTPSGSLVSSDAVLFNRPYWLERAQGQNNGILWNNDLFVTVLDNTRGTHFSISIATQDENDYTASNYKQYTRHVEEFELEFIFQLVKINLSTEVLAYLHGMDPSILDNWNLTLGPPNDGSLADKYRFIESLATKCPDNVEVTKPDPYKGRIFWNIDLTERLTADLDQFSLGRKFLYQHARISNRKRSLPASRNGGGTSSSSTKRRKK |
| 26 | 0.14 | 3 | GXXATGG | 2265 | 2768 | 168 | Yes | MAIGPKAYFYKDISSISVVPEESIELSTYTSAAPLGEDAGIIVEDSMEGSFDNITLSSWSHGSMDGLLEDDASYDFHGHLVWGTRRSSKQISMPFRRSWYPETAVYVQEGGSVMDPEASAELVPSRDSARPHVIYRGYNGTDYYLHPSLSRRRRKRRHIYFSDGVLAA |
| 30 | 0.13 | 3 | tXXATGG | 2454 | 2768 | 105 | Yes | MDGLLEDDASYDFHGHLVWGTRRSSKQISMPFRRSWYPETAVYVQEGGSVMDPEASAELVPSRDSARPHVIYRGYNGTDYYLHPSLSRRRRKRRHIYFSDGVLAA |
| 46 | 0.13 | 1 | cXXATGt | 3316 | 4248 | 311 | Yes | MSGMIEDGDMGDIGYGNLDFRVLQENKSEVPLEVVDSICKYPDYLGMSKETHGNSCFFYARREQLYSRHFFNRAGVQGETVPESLYKKGKDGQAQSTLALATYSGTPSGSLVSSDAVLFNRPYWLERAQGQNNGILWNNDLFVTVLDNTRGTHFSISIATQDENDYTASNYKQYTRHVEEFELEFIFQLVKINLSTEVLAYLHGMDPSILDNWNLTLGPPNDGSLADKYRFIESLATKCPDNVEVTKPDPYKGRIFWNIDLTERLTADLDQFSLGRKFLYQHARISNRKRSLPASRNGGGTSSSSTKRRKK |
| 45 | 0.12 | 1 | cXXATGt | 3211 | 4248 | 346 | Yes | MFIVGCVPAQGEHWSRALTCSNQVVKKGDCPPIQRMSGMIEDGDMGDIGYGNLDFRVLQENKSEVPLEVVDSICKYPDYLGMSKETHGNSCFFYARREQLYSRHFFNRAGVQGETVPESLYKKGKDGQAQSTLALATYSGTPSGSLVSSDAVLFNRPYWLERAQGQNNGILWNNDLFVTVLDNTRGTHFSISIATQDENDYTASNYKQYTRHVEEFELEFIFQLVKINLSTEVLAYLHGMDPSILDNWNLTLGPPNDGSLADKYRFIESLATKCPDNVEVTKPDPYKGRIFWNIDLTERLTADLDQFSLGRKFLYQHARISNRKRSLPASRNGGGTSSSSTKRRKK |
| 24 | 0.12 | 3 | GXXATGG | 2073 | 2768 | 232 | Yes | MGPTYINPAFEAEDSILFPESSMQAANPDYTGITRLGHLFGTEQGGRVRIGRLGQKTSLHTRSGMAIGPKAYFYKDISSISVVPEESIELSTYTSAAPLGEDAGIIVEDSMEGSFDNITLSSWSHGSMDGLLEDDASYDFHGHLVWGTRRSSKQISMPFRRSWYPETAVYVQEGGSVMDPEASAELVPSRDSARPHVIYRGYNGTDYYLHPSLSRRRRKRRHIYFSDGVLAA |
| 62 | 0.11 | 1 | GXXATGG | 3928 | 4248 | 107 | Yes | MDPSILDNWNLTLGPPNDGSLADKYRFIESLATKCPDNVEVTKPDPYKGRIFWNIDLTERLTADLDQFSLGRKFLYQHARISNRKRSLPASRNGGGTSSSSTKRRKK |
| 28 | 0.10 | 3 | tXXATGG | 2403 | 2768 | 122 | Yes | MEGSFDNITLSSWSHGSMDGLLEDDASYDFHGHLVWGTRRSSKQISMPFRRSWYPETAVYVQEGGSVMDPEASAELVPSRDSARPHVIYRGYNGTDYYLHPSLSRRRRKRRHIYFSDGVLAA |
| 41 | 0.10 | 2 | AXXATGG | 2912 | 3064 | 51 | Yes | MGILLLFLRSVLINTGCSVVNYRTLTGLHLVRSRFTTLRSNGLHGVYGEWR |
| 47 | 0.10 | 1 | GXXATGa | 3325 | 4248 | 308 | Yes | MIEDGDMGDIGYGNLDFRVLQENKSEVPLEVVDSICKYPDYLGMSKETHGNSCFFYARREQLYSRHFFNRAGVQGETVPESLYKKGKDGQAQSTLALATYSGTPSGSLVSSDAVLFNRPYWLERAQGQNNGILWNNDLFVTVLDNTRGTHFSISIATQDENDYTASNYKQYTRHVEEFELEFIFQLVKINLSTEVLAYLHGMDPSILDNWNLTLGPPNDGSLADKYRFIESLATKCPDNVEVTKPDPYKGRIFWNIDLTERLTADLDQFSLGRKFLYQHARISNRKRSLPASRNGGGTSSSSTKRRKK |
| 29 | 0.08 | 1 | GXXATGG | 2446 | 2535 | 30 | Yes | MDPWTGFLRMMLVMIFTATWCGEHAVALSK |

**Help for results table** (based on https://atgpr.dbcls.jp/table_help.html)

| No of ATG from 5' end | The number of an ATG's occurrence relative to the 5' end. |
| --- | --- |
| Reliability | Although the program works using the score from a linear discriminant function this is difficult for users to interpret. So, instead of showing the user the ldf score, it calculates a reliability score that states the number of times predictions are normally correct when a particular ldf score has been achieved. Specifically, the estimate of reliability derives from tests using a non-redundant set of 660 sequences.  For example, a reliability of 0.12 means that in tests the reliability of the ldf score was only 12% and therefore should be treated with the upmost caution. |
| Frame | Tells the user in which of the three possible frames each ATG was located. |
| Identity to Kozak rule | Kozak was the first to note that real initiation codons have a strong preference for the pattern A/GXXATGG. This section of the table shows the user how close a predicted ATG is to this pattern. |
| Start (bp) | The number of the nucleotide at which the translation starts (A of the initiating ATG). |
| Finish (bp) | The number of the nucleotide at which the translation finishes. This could be the end of the input sequence. See Stop codon found? |
| ORF length (aa) | The length of the predicted ORF in amino acids. This is (Finish-Start)/3. |
| Stop codon found? | Tells the user whether a termination codon was found or whether the program reads through to the end of the input sequence. |
